# Enhancing orthogonal finishing machining of Ti6Al4V with laser-ablated tool geometry modifications

**DOI:** 10.1007/s00170-024-14583-8

**Published:** 2024-10-28

**Authors:** Fabian Kneubühler, Nanyuan Zhang, Livia Haudenschild, Hagen Klippel, Matthias Putzer, Varun Urundolil Kumaran, Michal Kuffa, Konrad Wegener

**Affiliations:** 1 IWF, ETH Zürich, Leonhardstrasse 21, Zürich, Switzerland; 2 inspire AG, Technoparkstrasse 1, Zürich, Switzerland

**Keywords:** Ti6Al4V cutting, Laser modification, Laser micromachining, Micro-geometry, SPH simulation

## Abstract

Finishing machining of Ti6Al4V, known for its high strength and heat conduction resistance, demands optimisation to achieve high-quality end products. This study explores modifying the chip contact length on the rake face and altering the flank face with a cavity to minimise process forces and temperatures while maintaining cutting edge integrity. The research validates the manufacturability of ultra-short pulsed laser-ablated tool geometry modifications, indicating potential for industrial scale-up. Extensive experimental evaluations under dry conditions assess the impact of tool modifications at various feed rates for planing and turning. Significant reductions in process forces and temperatures were observed with rake face modifications, particularly at a cavity distance of approximately 34 µm. Ideal performance was noted for feed rates between 0.035 and 0.045 mm for planing and 0.040 to 0.045 mm/rev for turning. Smoothed Particle Hydrodynamics (SPH) simulations employing a Johnson-Cook material model were used to analyse chip formation and to predict the process forces. These simulations revealed a clear change in the chip formation and lower process forces and temperatures. The SPH results closely matched experimental outcomes, with a discrepancy of less than 7 % in cutting forces for both tool types, although feed forces were underestimated by about 50 %. The effect of the tool modification is reflected accurately at the respective feeds.

## Introduction

Titanium alloys, particularly Ti6Al4V, stand out with its high strength-to-weight ratio in combination with its good fatigue resistance at elevated temperatures and under corrosive conditions. These properties make Ti6Al4V the preferred choice for a variety of components of different systems in aerospace, medicine, chemical processing and the energy sector.

Cutting metals is a fundamental and indispensable process in manufacturing and its importance cannot be overstated. The cutting process allows the machining of complex shapes at high precision and accuracy using a wide range of techniques, e.g. planing, turning, drilling, milling, while offering scalability.

Even though Ti6Al4V has excellent properties it is more expensive than other materials like steel and aluminum. Moreover, its inherent characteristics, e.g. high strength, low thermal conductivity and chemical reactivity, characterise Ti6Al4V as a difficult-to-cut material. As a result, rapid tool wear, elevated process forces and poor surface finish are common challenges limiting its use from both engineering and economic perspectives. As a consequence, researchers and engineers have been continuously exploring techniques to overcome these limitations and improve the machinability of Ti6Al4V.

The cutting process is defined by the tool-workpiece interaction, which is significantly influenced by the tool geometry. The chip formation and development on the rake face within the chip contact length, connected with conditions on the flank face, cause large contact and frictional stresses that result in the respective process forces and process temperatures. According to the work of Zorev [1], the contact zone between the tool and the workpiece along the chip flow direction can be separated into a sticking, which is close to the cutting edge, and a subsequent sliding part. Both zones are associated with different friction, normal and tangential stresses. Zorev [1] states that tangential stresses are constant in the sticking region and exponentially decrease in the sliding region. In a recent work, [2] concluded that friction in the elastic contact region significantly affects contact length and feed force, while its impact is less pronounced in the plastic contact region. As a result, the authors suggest that rake-face textured tools lower process forces and enhance tool life. However, determining the chip contact length is challenging not to mention the location of the sticking and sliding zone. In [3], three different measurement approaches to determine the chip contact length are presented, namely the use of microtextured tools, in-situ visualisation and tools with restricted contact lengths. It is found that restricting the contact length reduces the process forces but also affects the contact length by itself. However, particularly for Ti6Al4V, the authors experienced difficulties to find the contact length because of the discontinuous chip formation that leads to varying chip thickness despite a constant uncut chip thickness. A rake angle ($\gamma$) of 0$^{\circ }$, a cutting edge radius (CER) of 20 µm, an uncut chip thickness of 0.1 mm and a minimum cutting speed ($v_c$) of 38 m/min was used, which is associated with rough machining. In this context, [4] showed that with decreasing $v_c$ and *f* and increasing $\gamma$, the segmentation degree decreases, where at $v_c$=80 m/min and $\gamma$=15$^{\circ }$ a very weak segmentation was found, which suggests that the challenges due to discontinuous chip formation described in [3] can be minimised.

On the flank face, spring back after deformation significantly affects the workpiece precision, with recovery influenced by factors such as elastic modulus, grain size, and deformation rate [5]. The spring back alters the contact between the cutting tool and the workpiece, affecting the distribution of the process forces and depending on the severity, increases the process forces, as exemplified by the machining of CFRP in [6]. While reducing the contact area on the flank face may help mitigate spring back effects in Ti6Al4V machining, it is important to note that these effects typically manifest within a small, single-digit micrometer range [7], which inherently limits contact areas on the flank face. Therefore, any noticeable impact would require very small distances of the cavity from the cutting edge.

In finishing machining, sharp tools with corresponding small feed rates and large rake angles are required to minimise the process forces and therefore produce finer and more accurate surface finishes, which is crucial for precision and high-quality finished products [8, 9]. Considering these boundary conditions, the literature does not provide information that reveals the reduction of process forces by altering the chip contact length on the rake face or the spring back on the flank face. This is explained by the fact that small feeds, e.g. in the range of *f*=0.010 to 0.050 mm, lead to small chip contact lengths in the same order of magnitude. From a manufacturing point of view, this places demands on accuracy in the lower single-digit micrometre range for position and alignment to the cutting edge. Consequently, the relationship between feed rate, cavity distance to the cutting edge, and potential force reduction from decreased contact at the flank face remains unclear.

For this work, tools with a uncoated cemented carbide substrate material with a rake angle $\gamma$=20$^{\circ }$, a clearance angle $\alpha$=7$^{\circ }$ and a cutting edge radius of approximately 4 to 8 µm is chosen to analyse the effect of tool modifications in dry orthogonal cutting experiments. Since the area of influence of the modification must be in the order of magnitude of the feed or spring back, high accuracy and small material removal are required to create effective tool modifications. Therefore, ultra-short pulsed laser-based techniques are a suitable technology to produce modified cutting tools satisfying the condition of small material removal and offer low thermal impact and the absence of process forces. For the laser machining of the modifications, the cutting edge and the respective tool surfaces need to be detected and aligned orthogonally to the laser beam. This is developed on a commercially available laser machine showing the potential for industrialisation of laser-ablated tool geometry modifications. A detailed experimental study reveals the process forces and temperatures for a rake and flank face-modified tool. In a subsequent step, a Smoothed Particle Hydrodynamics (SPH) method to model the orthogonal cutting process with a rake face cavity is applied and validated with the experimental results. In this context, a Johnson-Cook material model is incorporated and determined by an inverse parameter identification according to the methodology presented in [10]. The SPH simulation is used to model different process conditions to analyse the chip formation and to predict the outcome of the process forces.

## Manufacturing of laser-ablated tool geometry modifications

The goal is to produce rake or flank face-modified tools that restrict the contact length between the tool-workpiece interface, which allows for a 2D approximation of the orthogonal cutting processes for the numerical simulation. The following sections detail the setup, the laser processing strategy and the laser parameters.

**Fig. 1:**
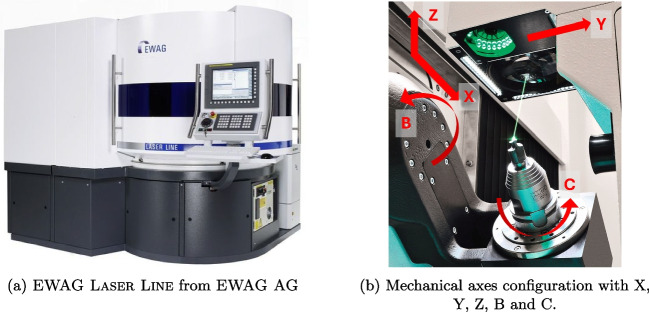
Laser machine for producing laser-ablated tool gemetry modifications

### Setup laser modification production

The laser production is performed on the laser machining centre type EWAG Laser Line from EWAG AG having a Fanuc control. The machine has 5 mechanical (X,Y,Z,B,C) and 3 optical axes (U,V,W). Figure 1 displays the machine and its mechanical axes configuration. The laser beam emits pulses in the picosecond regime (<15 ps), is near-infrared (1064 nm) and with the help of polarising plates is circularly polarised. Additionally, the laser beam shape and intensity distribution are circular and Gaussian respectively. With the help of a f-theta lens, the laser beam is focussed down to a diameter, $\omega _{0}$, of approximately 25 µm. The maximal average laser power $P_m$ is 50W with a pulse repetition rate $f_p$ of 400 to 2000 kHz. A Renishaw MP250 touch probe fixed to the Y-axis is installed and the software LaserSoft from EWAG AG is used to operate the machine.

### Calibration

The positional accuracy of the lasered structures on the tool faces is essential not only to achieve repeatable cutting results but is necessary owing to the applied small feeds on the cutting tools. For this reason, calibration of the machine tool is vital. Calibration entails the following 3 steps:

1. Elimination of the distortion resulting from the scanning system, inspired by [11].
2. Calibration of the laser beam zero point relative to the machine zero point.
3. Calibration of the measurement probe zero point relative to the laser zero point.

### Laser process strategy of tool modification

**Fig. 2:**
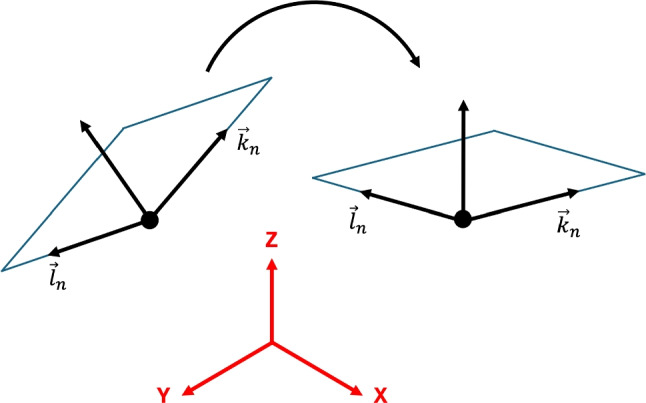
Alignment of the tool surface, described by the vectors $\textbf{k}_n$ and $\textbf{l}_n$, to be orthogonal to the laser beam, which aligns with the Z-axis of the global coordinate system

**Fig. 3:**
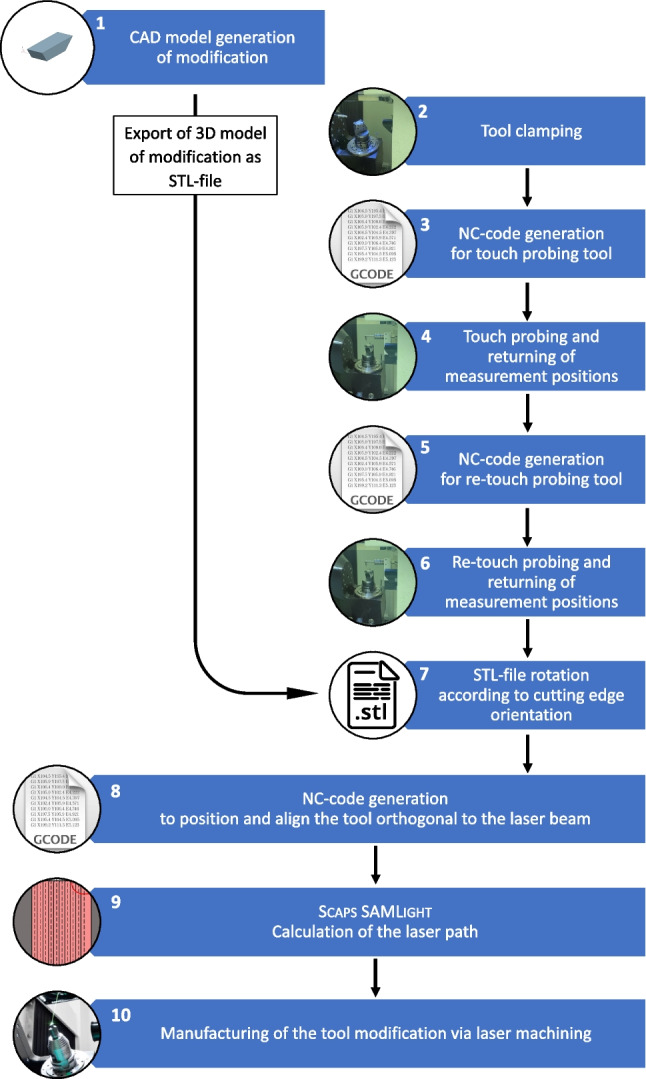
10-step process flow outlining the manufacturing of laser-ablated tool modifications using the Laser Line machine from EWAG AG to achieve single-digit micrometer positional and alignment accuracy

The steps to machine the laser modifications on cutting tools are visualised in the process flow shown in Fig. 3, which is detailed in the following section. In the beginning, a 3D model of the target ablation volume is generated, Fig. 3 (1), for which the CAD software NX from Siemens is used. For the laser process, the CAD model is exported as an STL-file for further processing. At the machine, the laser production starts with the clamping of the cutting insert in the tool holder, Fig. 3 (2). It is initially assumed that the surfaces to be machined, i.e. the rake and flank faces, are inclined. A measurement routine is applied to touch probe these surfaces to calculate a solution to the position of the mechanical axes necessary so that the initially inclined surface is transformed and orientated orthogonal to the laser beam. The measurement routine is an NC-code that is generated by a parametric script written in Matlab. Subsequently, LaserSoft orchestrates the control of the laser and scanning system together with the writing of the NC-Code into the Fanuc control system, Fig. 3 (3). The measurement coordinates are written to the Fanuc control and returned to Matlab, Fig. 3 (4), to calculate the axes position to align the respective tool face orthogonal to the laser beam, which is in Z-direction of the machine’s global coordinate system. The visualisation for a vector along the cutting edge, $\textbf{k}_n$, and a vector orthogonal to $\textbf{k}_n$ and along the respective tool face, $\textbf{l}_n$, is shown in Fig. 2.

Consequently, the calculation of the B- and C-axis positions such that the tool face lies in the machine’s X-Y-plane is required, which is detailed in Appendix A. Via rotation matrices and with the system of equation satisfying the condition that the Z-components are zero, the following solution from Eq. A7 for B and C is obtained as follows:

1 $$\begin{aligned} B= &   atan \left( \frac{-kz_n}{{\sin }(C) \cdot ky_n - {\cos }(C) \cdot kz_n} \right) \end{aligned}$$

2 $$\begin{aligned} C= &   atan \left( \frac{kx_n \cdot lz_n - lx_n \cdot kz_n}{ky_n \cdot lz_n - ly_n \cdot kz_n} \right) \end{aligned}$$

With the solutions of the B- and C-axis, the tool’s rake or flank face can be aligned orthogonal to the laser beam and the position of the vectors $\textbf{k}_{n+1}$ and $\textbf{l}_{n+1}$ are calculated using Eqs. A5 and A6 respectively. Since high accuracy in the one-digit micrometer scale is required, the tool’s surface needs to be re-touch probed in the aligned state in order to minimise machine axes and touch probe errors, e.g. magnification of angular errors over distance (Abbe error) and owing to errors arising to the contact position on the spherical measurement probe. Consequently, a new NC-code is generated to re-touch probe the cutting edge, Fig. 3 (5), to measure the remaining offsets of the cutting edge position. The measurement positions are again written to the Fanuc control and returned to Matlab, Fig. 3 (6). The exported STL-file from Fig. 3 (1) is opened with Matlab, rotated based on the cutting edge vector in the X-Y plane measured in Fig. 3 (6), and exported to the laser machine, Fig. 3 (7). In step Fig. 3 (8), the NC-code is generated to position and align the cutting tool’s processing face orthogonal under the laser beam. The laser path of the STL-file is calculated by the software Scaps SAMLight, Fig. 3 (9). In the last step Fig. 3 (10), the modifications are machined on the tool’s surface by the laser through material ablation.

### Laser parameter

Based on the hardware specifications of the used laser machine, detailed in Section 2.1, and reference values from the literature, the laser parameters are determined. The pulse overlap (*PO*) is calculated as follows:

3 $$\begin{aligned} PO = \left( 1-\frac{v_s}{2 \cdot \omega _0 \cdot f_p}\right) \ \ [\%] \end{aligned}$$

where $v_s$ is the scanning velocity, $\omega _0$ the circular focus radius and $f_p$ the pulse repetition rate. The scanning system is set to be fixed to $v_s$=1 m/s. For orthogonal laser ablation of cemented carbide substrate materials, [12] showed that pulse overlaps from 50 to 90 % are suitable. It is concluded that larger pulse overlaps increase the surface roughness and decrease the hardness in the surface layer, however, no significant effect on the residual stresses was found. As a result, $f_p$ is set to 400 kHz leading to a *PO* of 90 %. The fluence, denoted by *F*, represents the energy delivered by the laser per unit area and is defined in:

4 $$\begin{aligned} F = \frac{2P_m}{\pi f_p {\omega _0}^2} \ \ [\textrm{J}/\textrm{cm}^{2}] \end{aligned}$$

where $P_m$ is the average laser power. According to [13], the single-pulse ablation threshold of cemented carbide is independent for the tested pulse durations of 0.2 to 10 ps at a wavelength of 1030 nm and is about 0.4 J/cm$^{2}$. For optimal outcomes in lasered structures, a material removal rate between 5 and 15 times higher than the ablation threshold fluence is recommended [14]. This range is associated with efficient material removal, contributing to enhanced surface quality and precision. In [12], suitable fluences for cutting edge preparations of cemented carbide inserts were found to be between 1.4 J cm$^{-2}$ and 4.2 J cm$^{-2}$.

For this work, $P_m$ is set to 3.9 W, which results in *F*=4.0 J/cm$^{2}$. The parameters for laser processing are summarised in Table 1. To calculate the laser path mentioned in Section 2.3, the 3D shape of the modification is divided into several layers of flat processing surfaces. This requires the ablation depth per ablated layer. It is determined using a laser machined pocket with a size of 100$\times$100 µm. The 3D shape of the laser-machined pocket is measured with a confocal microscope and the depth is determined, which is 50 µm. With a number of 83 ablation layers, this results in an ablation depth of 0.6 µm per layer.

**Table 1:** Laser parameter settings to machine cemented carbide cutting inserts

*F*	$f_p$	*PO*	$P_m$	$v_s$	*h*
4.0 J cm$^{-2}$	400 kHz	90 %	3.9W	1 m s$^{-1}$	0.6 µm

*F*: fluence; $f_p$: pulse repetition rate; *PO*: pulse overlap; $P_m$: average laser power; $v_s$: scanning speed; *h*: layer height

## Experimental methodology

In this section, the material and methods for the cutting experiments, i.e. planing and turning, are presented.

### Workpiece material

The Ti6Al4V workpiece material for the planing experiments is hot-rolled to a thickness of 6 mm, annealed and pickled, and then cut via water-jet to a dimension of 200 mm length and 30 mm width. The mechanical properties of the material are determined by non-destructive and non-contact measurements of its resonant frequencies using the EMATRUS technology developed by EMATronics. This technology enables the precise and rapid determination of critical mechanical properties such as the modulus of elasticity (Young’s modulus), shear modulus, and Poisson’s ratio. Two specimens, specimen 35 aligned with the rolling direction (RD) parallel to the cutting speed and specimen 44 aligned orthogonally, are analysed. Specimen 35 aligns the X-axis with RD, while for specimen 44, the X-axis aligns with the 90$^{\circ }$ RD. It is found that the Young’s modulus varies between 111 and 129 GPa resulting in $\approx$ 14 % difference as shown in Table 2.

**Table 2:** Comparison of mechanical properties between specimen 35, aligned parallel to the cutting speed, and specimen 44, oriented orthogonally to the rolling direction

Property	Specimen		Units
	35	44	
Density $\rho$	4393	4389	kg m$^{-3}$
Modulus $E_x$	110.6	129.4	GPa
Modulus $E_y$	117.6	121.7	GPa
Poisson’s ratio $v_{xy}$	0.3366	0.3521	−
Shear modulus $G_{xy}$	40.72	42.96	GPa

**Table 3:** Chemical analysis of Ti6Al4V workpiece specimens using an Optical Emission Spectrometer (OES), which measures the material composition, expressed as percentages by weight

Spec	Meas	Al	C	Cr	Fe	Mn	V	Mo	Ni	Si
	1	5.93	0.009	0.005	0.16	<0.005	4.13	<0.015	<0.002	0.004
35	2	5.97	0.009	0.005	0.16	<0.005	3.88	<0.015	<0.002	0.007
	3	5.95	0.009	0.005	0.16	<0.005	3.92	<0.015	<0.002	0.004
	*Avg.*	*5.95*	*0.009*	*0.005*	*0.16*	<*0.005*	*3.98*	<*0.015*	<*0.002*	*0.005*
	1	5.88	0.009	0.005	0.17	<0.005	3.89	<0.015	<0.002	0.010
44	2	5.95	0.009	0.005	0.17	<0.005	3.98	<0.015	<0.002	0.008
	3	5.93	0.010	0.005	0.16	<0.005	3.95	<0.015	<0.002	0.011
	*Avg.*	*5.92*	*0.009*	*0.005*	*0.17*	<*0.005*	*3.94*	<*0.015*	<*0.002*	*0.010*

Three measurements (Meas) are performed and averaged for each specimen. Specimen (Spec) 35 is aligned with the rolling direction parallel to the cutting speed, and Spec 44 orthogonally

**Fig. 4:**
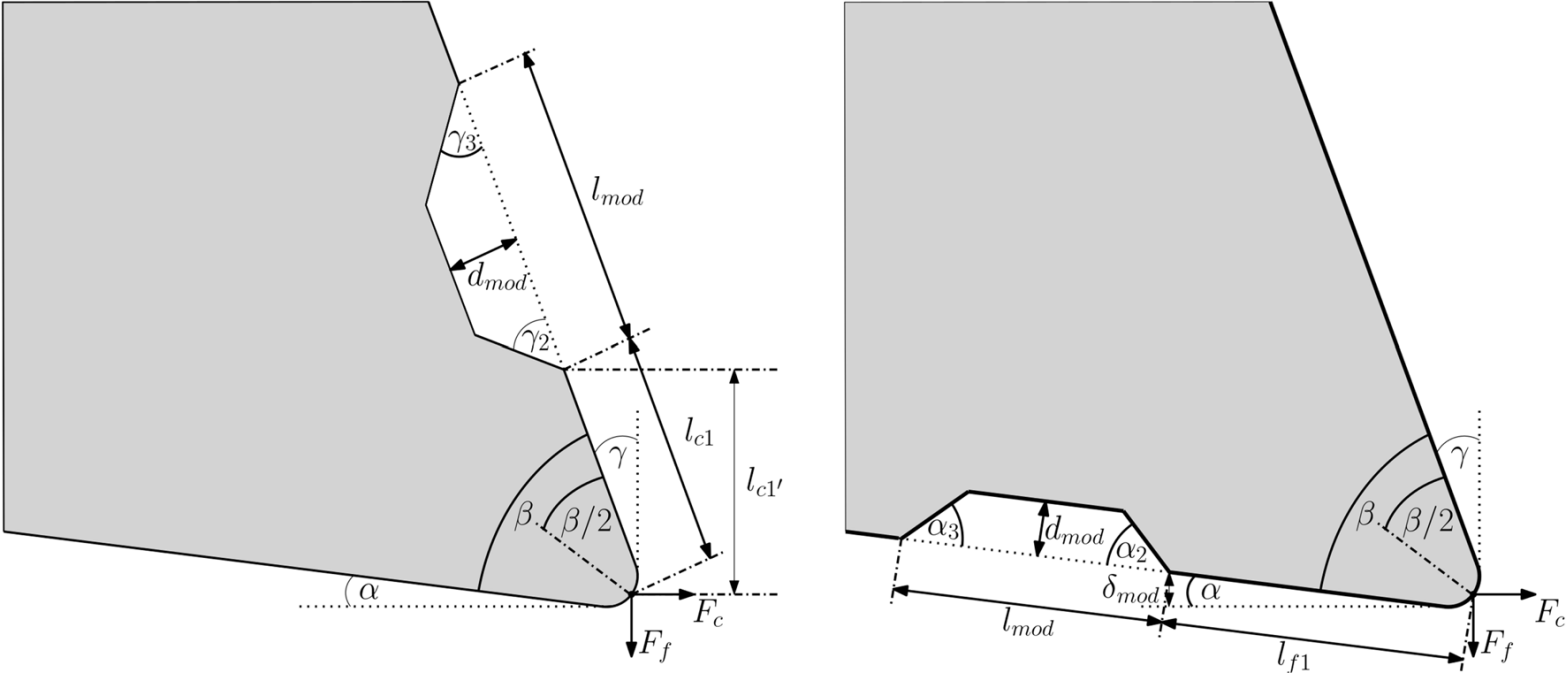
Schematic of a rake face (left) and flank face (right) modified tool, where the modifications are described by $l_{c1}$ or $l_{f1}$, $\gamma _2$ or $\alpha _2$, $d_{mod}$, $\gamma _3$ or $\alpha _3$, and $l_{mod}$. The projection of $l_{c1}$ in feed force $F_f$ or orthogonal to the cutting force $F_c$ direction is called $l_{c1'}$. $\delta _{mod}$ indicates the position of the modification in the direction of the elastic recovery, referred to as spring back, i.e. $F_f$ direction. The modification is referred to as Täschli (Swiss German for small pocket). The rake angle $\gamma$ and the clearance angle $\alpha$ define the tool’s macro geometry

Optical Emission Spectrometer (OES) measurements are employed to analyse the material composition. According to [15], the method involves exciting atoms in a sample and measuring the distinct wavelengths of light emitted, which correspond to different elements. These measurements are conducted using the SPECTROMAXx device by Spectro Ametek at the Institut für Mikrotechnik und Photonik of the Ostschweizer Fachhochschule (OST). The analysis indicates that the two specimens are less than 0.1 % by weight similar as detailed in Table 3, confirming they originate from the same batch despite differences in Young’s modulus shown in Table 2.

Based on previous cutting experiments, a cutting width of 6 mm leads to process vibrations and cutting edge instabilities. Consequently, the thickness is reduced by cutting the workpiece in half using wire electric discharge machining (WEDM). Due to residual stresses in the workpiece, the thickness varies after the WEDM, which is corrected by a subsequent milling operation. Since the thickness value between different workpiece stripes is subject to deviations of $\approx$ 0.2 mm, every workpiece is measured via a height gauge to normalise the force measurements to a constant thickness of 1 mm. Cutting experiments are performed with an approximate workpiece thickness of 2.5 mm.

For the turning experiments, a hollow cylinder is used, with an outer diameter of approximately 200 mm and an initial wall thickness of $\approx$ 7 mm, which is machined before the experiments to $\approx$ 2 mm wall thickness. The hollow cylinder is not from the same material batch as the stripes for the planing experiments. Besides the material supplier’s certificate accompanying the batch, no specific studies on mechanical properties or chemical composition have been conducted for this material batch. To estimate the influence of the material batch between a cylindrical and a stripe workpiece, planing experiments were conducted using a different cylinder that allowed to be cut to a stripe. The cylindrical raw material is characterised in [10], exhibited isotropic properties with a yield strength of 867 MPa. This batch was compared with the rolled strips mentioned in the begining of this section, which exhibited anisotropic properties, showing a 15.5 % higher yield strength in the $90^{\circ }$ direction compared to the $0^{\circ }$ direction, reaching 970 MPa. The experiments revealed a 12 % higher feed force $F_f$ in the 90$^{\circ }$ direction, while the cutting force remained similar. The variations between batches were less significant than the anisotropic effects within batch of the cylindrical material, consistent with material testing results.

### Tools

The sintered uncoated cutting inserts have a ground clearance angle of $\alpha$=7$^{\circ }$ and rake angle of $\gamma$=20$^{\circ }$ and no further cutting edge preparation. The cemented carbide substrate material EMT100 from the supplier EXTRAMET consists of an average submicron grain size of 0.8 µm and a 6 % cobalt content.

The laser-ablated tool geometry modifications are produced according to the methodology described in Section 2. A Täschli (Swiss German for small pocket) is ablated in the respective tool face, where a schematic of rake and flank face modified tool is shown in Fig. 4. The defining parameter of the location is the distance $l_{c1}$ on the rake face or $l_{f1}$ on the flank face between the beginning of the modification and the furthest point of the cutting edge, which lies in the bisector of the wedge angle $\beta$. This length depends on the micro geometry of the cutting edge, specifically on the edge flattening parameter $\Delta r$, introduced in Section 3.4. Additionally, $l_{c1}$ needs to be considered with respect to the feed force $F_f$ and the cutting force $F_c$, since the chip contact length geometrically depends on $\gamma$. Consequently, $l_{c1'}$ is given as follows:

5 $$\begin{aligned} l_{c1'} = {\cos }(\gamma ) \cdot l_{c1} \end{aligned}$$

On the flank face, the modification position is regarded in the $F_f$ direction of the elastic recovery of the material, i.e. spring back height, which results in $\delta _{mod}$:

6 $$\begin{aligned} \delta _{mod} = {\sin }(\alpha ) \cdot l_{f1} \end{aligned}$$

**Table 4:** Measured geometry parameters of laser-modified tools

Tool	Mod	$l_{c1}$, $l_{f1}$	$l_{c1'}$, $l_{f1'}$	$\delta _{mod}$	$\gamma _2$, $\alpha _2$	$\gamma _3$, $\alpha _3$	$d_{mod}$	$l_{mod}$
	face	[µm]	[µm]	[µm]	[°]	[°]	[µm]	[µm]
T529_L1_A	Rake	36.4	34.2	−	31	50	46	193
T529_L1_B	Rake	35.9	33.7	−	31	50	46	193
T534_L1_A	Flank	33.2	32.9	4.0	26	22	29	250

The tool designation includes the tool number, followed by the tool side, the cutting edge number, and the position on this edge. $l_{c1}$ or $l_{f1}$ describes the position of the modification on their respective face with respect to the cutting edge and $l_{c1'}$ is the projected distance orthogonal to the cutting or parallel to the feed force direction. $\delta _{mod}$ indicates the position of the modification in the direction of the elastic recovery, referred to as spring back, i.e. $F_{f}$ direction. $\gamma _2$ or $\alpha _2$, $\gamma _3$ or $\alpha _3$, $d_{mod}$ and $l_{mod}$ characterise the geometry of the modification as the inlet angle, the outlet angle, the depth and the length

The geometry parameters of the Täschli are the inlet angle into the modification $\gamma _2$ or $\alpha _2$, the depth of the modification $d_{mod}$, the outlet angle $\gamma _3$ or $\alpha _3$ and the length of the modification $l_{mod}$.

The geometric parameters of the laser-modified tools are summarised in Table 4, as measured using a Sensofar S Neox microscope. A target value of $l_{c1}$ or $l_{f1}$ of 35 µm is achieved within ±2 µm. The 3 measured distances to the cutting edge are all within 4 µm. The tool designation consists of a tool number, i.e. 529 and 534, followed by the specification of the tool side, i.e. left, the respective number of the cutting edge on the tool, i.e. 1, and the position on this cutting edge, i.e. A or B, where A is used for planing and B for turning.

**Fig. 5:**
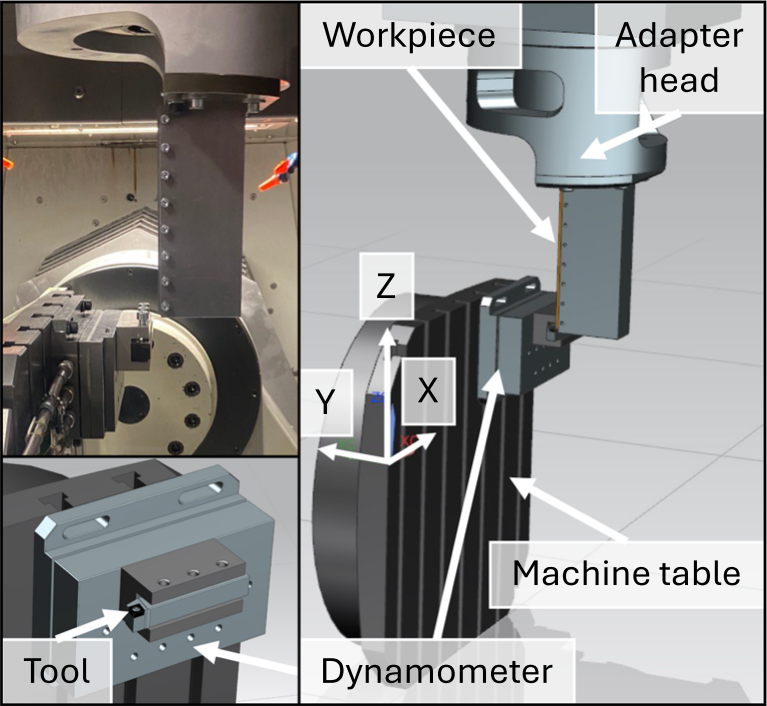
Schematic of the planing test bench showing on the left bottom the close-up view of the tool mounted on the dynamometer, which is fixed to the machine table. On the right side, the overview of the setup with the dynamometer, tool and workpiece fixed to the adapter head, is displayed. The adapter head is connected to the machine’s *Z*-axis. The cutting movement is in *Z*-direction and the feed movement is in Y. On the top left is the orthogonal cutting view of the actual setup

**Fig. 6:**
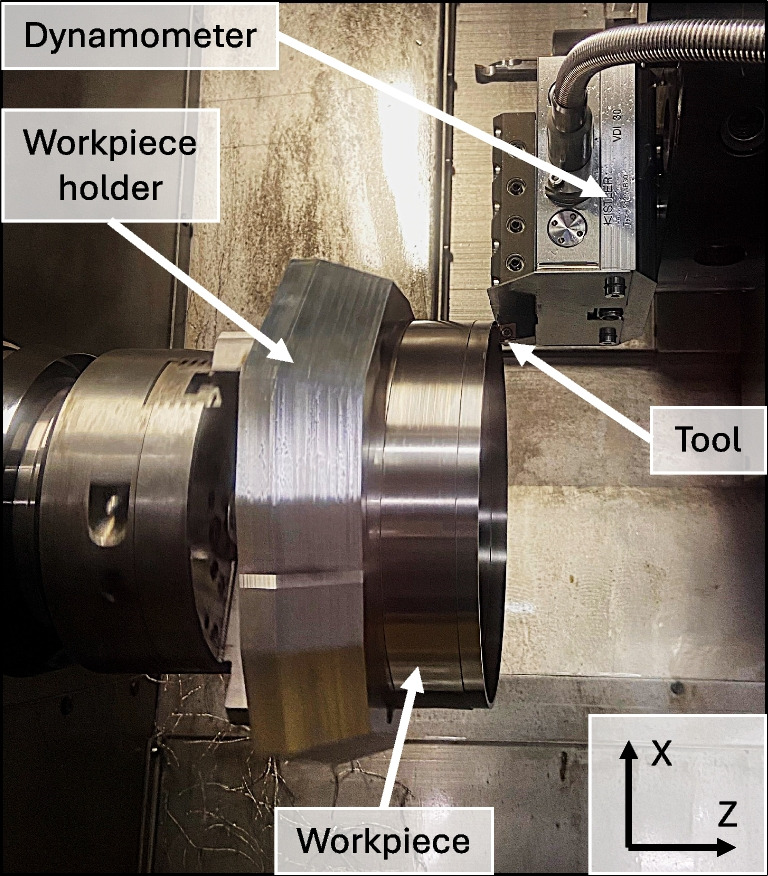
Turning test bench showing the tool mounted on the dynamometer and the workpiece fixed to the workpiece holder

### Experimental setup

Planing (orthogonal) and turning (quasi-orthogonal) cutting is used to evaluate the laser-modified tools and to study the difference between these two process kinematics.

The orthogonal cutting experiments are performed on a self-developed planing test bench. Planing is defined in DIN 8589 [16]. In this setup, the cutting and feed movement are performed with the workpiece, while the tool is fixed at one position facilitating the measurement of the process forces and temperatures. The test bench is set up on a Fehlmann Versa 825 CNC milling machine, where the force measurement platform and the tool are fixed on the machine table and the workpiece is mounted on the machine’s Z-axis by an adapter head shown in Fig. 5. A multi-component dynamometer of the type 9257B from Kistler with an industrial 1-4-channel charge amplifier of the type 5073A from the same manufacturer is used to obtain the process forces. The feed movement perpendicular to the cutting direction is executed by the Y-axis.

For turning, a hollow cylinder is clamped with a three-jaw chuck and a self-developed workpiece holder on a Schaublin 42 L turning lathe, as illustrated in Fig. 6. In this setup, the workpiece rotates around the Z-axis to perform the cutting movement, while the tool executes the feed movement in the Z-direction. Process forces during turning experiments are measured using a type 9129A piezoelectric multicomponent dynamometer and a type 5165A charge amplifier, both supplied by Kistler. The bulk raw material was machined before the experiments on the outer and inner diameter in order to reduce the thickness to $\approx$ 2 mm for minimising the load on the tool, the cutting speed variations between inner and outer diameter and the roundness errors.

### Data evaluation

In this section, the data evaluation methods for the cutting edge and the process forces are presented. Experimentally obtained data do not necessarily follow a Gaussian distribution. As a consequence, using the standard deviation as the sole measure of variability may not accurately reflect the true spread of the data. Therefore, quartiles are calculated, which are defined as follows:

- Q1 (first quartile / lower quartile): 25^th^ percentile (lowest 25 % of data)
- Q2 (second quartile / median): 50^th^ percentile (cuts data set in half)
- Q3 (third quartile / upper quartile): 75^th^ percentile (highest 25 % of data)

The advantages, among other things, are robustness to outliers, representation of non-normal distributions and robustness to skewness and interpretability.

In order to be able to compare the force results from turning and planing, the process forces in turning are divided in 0.2 m cutting length intervals, which corresponds with the cutting length of a planing stroke. These intervals are evaluated by means of the statistical parameters Q1, Q2 and Q3.

The differences of the forces and temperatures are analysed by their percentage differences, exemplarily calculated using the force, but applied analogously for the temperature:

7 $$\begin{aligned} \Delta F(f_i) = \frac{F_{mod}(f_i) - F_{ref}(f_i)}{F_{ref}(f_i)} \cdot 100 \ [\%] \end{aligned}$$

where $f_i$ refers to the present feed. *F* stands for the respective force component, i.e. $F_c$ and $F_f$, while $F_{ref}$ indicates the force from the reference tool and $F_{mod}$ the modified tool. $\Delta F(f_{i})$ is negative, when the modification results in a process force reduction. In order to quantify the changes between the percentage deviations from Eq. 7, the gradient, denoted $\Delta F'(f_i)$, is calculated as follows:

8 $$\begin{aligned} \Delta F'(f_i) = \frac{\Delta F(f_i) - \Delta F(f_{i-1})}{f_{i} - f_{i-1}} \ \left[ \frac{\%}{\mu m}\right] \end{aligned}$$

which calculates the difference between the percentage difference at feed i and i - 1. This formula represents the slope of the deviation changes with respect to the variable *f*, indicating the sensitivity of deviations to changes in *f*. For this work, $f_{i} - f_{i-1}$ remains constant at 5 µm.

For the evaluation of the chip cross-sections, the automated analysis methodology from [17] is applied. The methodology uses image processing techniques to extract various chip parameters, where for this work, only the chip thickness is taken into account.

#### Cutting edge characterisation

**Fig. 7:**
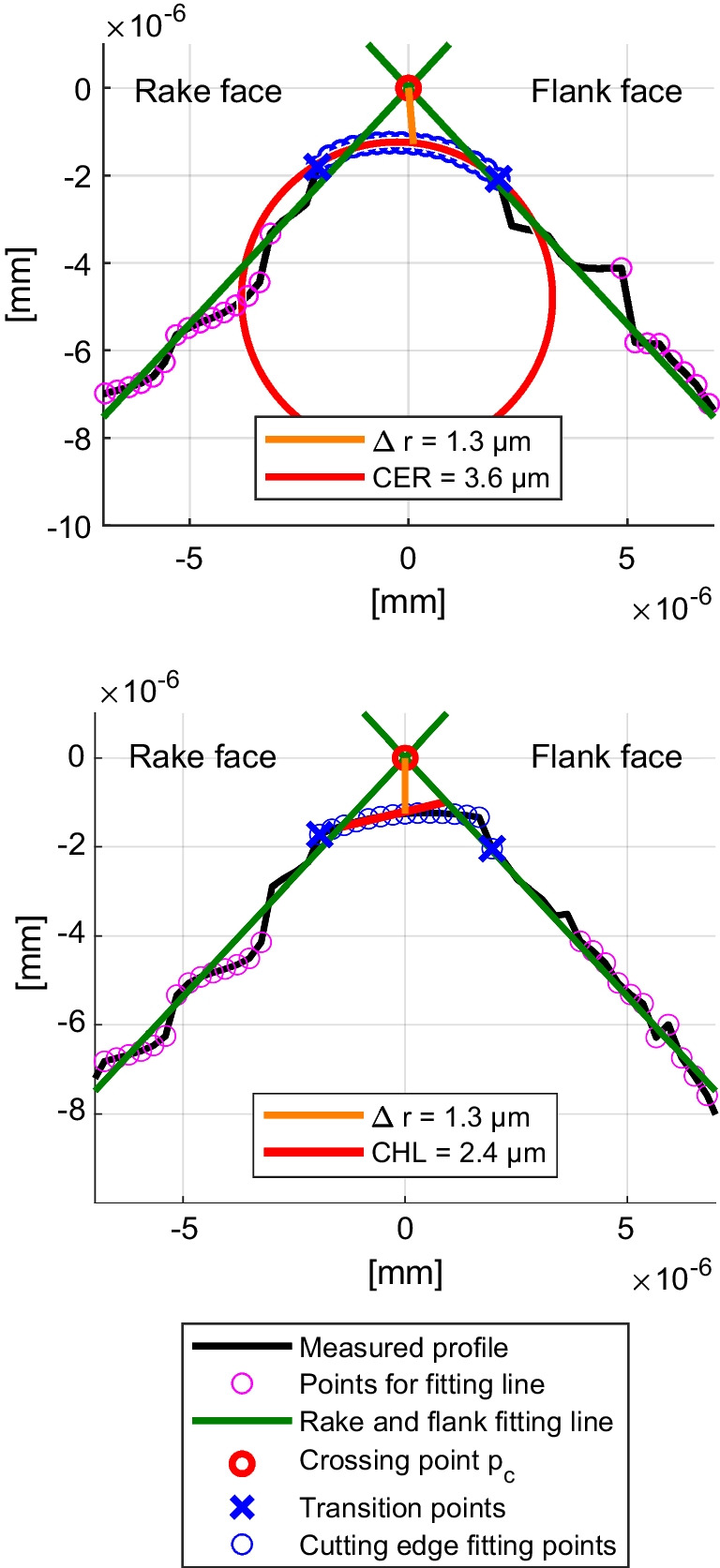
Characterisation of the cutting edge according to Wyen’s algorithm [9]. Initially, points on the rake and flank face (pink) are used to generate a least squares straight line fitting (green) in order to determine the transition points (blue crosses) to the cutting edge and to create the intersection point of an ideally sharp tool. The edge flattening $\Delta r$ (orange) is found by the intersection point between rake and flank face and the point on the tool following the bisector of the wedge angle. The points representing the cutting edge (blue circles) are distinct between fitting a cutting edge radius (CER) (top) or a chamfer length (CHL) (bottom)

The characterisation of a cutting edge depends on the tool’s geometry, the tool’s surface properties, the data acquisition procedure, the post-processing of the data and the evaluation methodology. In this work, a Sensofar S neox 5 Axis microscope is used to obtain a 3D point cloud of the cutting edge. The technologies integrated in the microscope are focus variation with active illumination and confocal microscopy, which are used interchangegebly to get the best data quality. Active illumination improves focus positioning on smooth surfaces, i.e. ground or worn cutting tools. Confocal measurement results in the highest lateral resolution. A 50X EPI CF60-2 Nikon objective lense is used resulting in a lateral resolution of 0.13 µm. A representative excerpt of the 3D point cloud of the cutting edge of 1.5 mm length is cropped resulting in $\approx$ 5000 cutting edge profiles. The 3D point cloud is split into 2D profiles along the cutting edge, and each profile of the cutting edge is characterised by applying Wyen’s algorithm [9]. At the beginning, the algorithm iteratively determines the transition points, shown as blue crosses in Fig. 7, between the rake and flank faces. To achieve this, points on the rake and flank faces (pink circles) are used to calculate a least squares fitting line (green), which is necessary to find the points representing the cutting edge (blue circles). Afterwards, a cutting edge radius (CER) is determined by generating a least squares circle using all the points within the previously found transition points. As a consequence of this definition, the center of the circle does not necessarily need to be on the wedge angle bisector or the fitted circle tangent to the rake and flank face. If the quality of fit is poor, i.e. the measured points cannot be sufficiently represented by a circle, Wyen considers a line fitting resulting in the description of a chamfer length (CHL) instead of a CER, displayed in Fig. 7 on the bottom. In addition to the description of the approximated shape of the cutting edge, i.e. CER or CHL, the parameter edge flattening $\Delta$r is determined as the distance between the intersection point $p_c$ of an ideally sharp tool and the point on the cutting edge along the wedge angle bisector, visualised with a vertical orange line for CER and CHL in Fig. 7. The edge flattening takes into account that larger cutting edge radii do not necessarily indicate a non-pointy tool, which is especially relevant for small feeds that are in the order of magnitude of the CER. However, $\Delta$r is dependent on the wedge angle $\beta$ and the CER so that for different wedge angles and cutting edge radii, the same $\Delta$r can be obtained. Therefore, this work characterises the cutting edge using the CER, CHL and $\Delta$r, hereinafter referred to as cutting edge characterisation parameters (CECPs), simultaneously in order to account for their respective advantages and disadvantages and to accurately describe the tool-workpice interface. In addition to the quantitative characterisation, the cutting edge including the active micro geometry that is involved in the tool-workpiece interaction is assessed for cutting edge breakouts.

#### Process forces

**Fig. 8:**
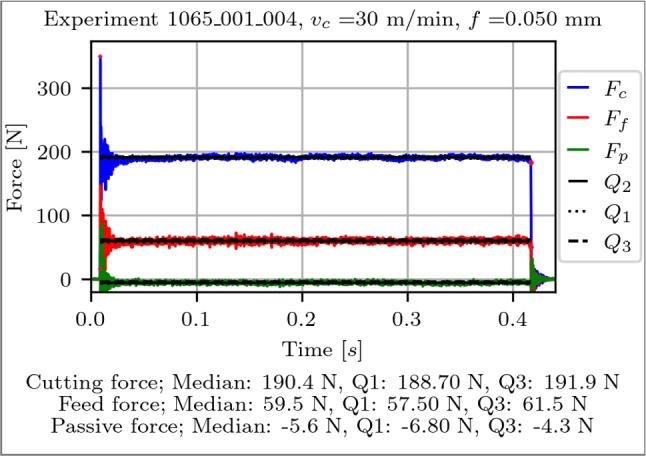
Exemplary evaluation of the cutting (blue), feed (red) and passive (green) force over time of a single orthogonal planing stroke, where a linear drift compensation was performed beforehand to offset the signal start and end of the signal rise to zero. The computed Q1, Q2 and Q3 values are visualised for each force component respectively

Since dynamometer force measurements can be subject to gradual change in the measured quantity over time, e.g. due to parasitic currents, a drift compensation is applied to each measurement. The duration of each stroke is approximately 0.4 s. An idle force is calculated as the mean value over a 0.5 s period, approximately 0.1 s before and after each stroke, to perform a linear drift compensation and to offset the signal at the beginning and end to zero. After the compensation, the start and end points of the signal evaluation are determined iteratively, starting from the signal rise and continuing until the first point of decrease is identified. Then, Q1, Q2 and Q3 are computed. The visualisation of a processed signal is shown in Fig. 8.

#### Process temperatures

**Fig. 9:**
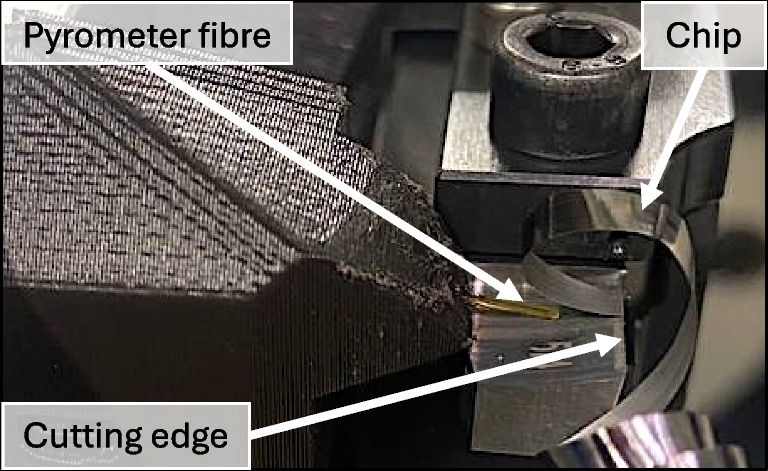
Temperature measurement setup on planing test bench showing the pyrometer fibre located on the tool’s rake face behind the chip’s free face pointing towards the cutting edge. The chip is adheared to the cutting edge after the experiment

A fast fiber-optic two-color pyrometer, Fire-3, is used for temperature measurements on surfaces with varying and unknown emissivities. It features a 0.5 mm fiber diameter with a polyimide thermocoat. The device is able to measure temperatures between 200 and 1200 $^{\circ }$C with a maximum sample rate of 500 kHz. For this work, a sample rate of 10 kHz is chosen resulting in a time resolution of 0.1 ms. The pyrometer development and further information are found in [18]. The flexible fiber allows to be placed orthogonal to the cutting edge as shown in Fig. 9.

**Fig. 10:**
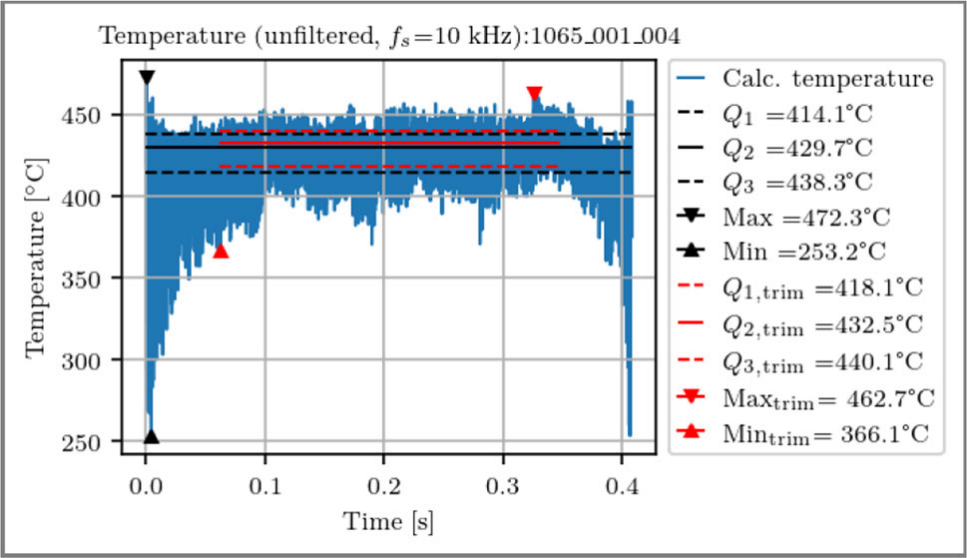
Exemplary evaluation of the process temperature (blue) over time of a single planing stroke. The computed statistical parameters are displayed for the entire signal duration (black) and for the segment cropped by 15 % at the start and end of the signal (red)

Evaluation boundaries are established, and temperatures are calculated based on the ratio of the channel outputs. The signal evaluation area is defined to exclude 15 % of the signal duration at the start and end, corresponding to approximately 0.12 s, to mitigate effects from running-in behavior. Subsequently, the statistical parameters of the data are computed in this trimmed time range. An example of a temperature measurement is illustrated in Fig. 10.

## Experimental results

This section presents the experimental results of the feed studies, which examine the performance of laser-modified tools across various feeds or feed rates, incrementally increasing from *f*=0.010 to 0.050 mm or mm/rev in 5 µm steps. The analysis begins with a detailed characterisation of the cutting edge of the tools used. Following this, the resulting process forces, i.e. $F_c$ and $F_f$, and the process temperatures *T* are analysed and discussed for both planing and turning. A comparison is then provided to highlight the performance differences observed in these two orthogonal process kinematics, emphasising the importance of diverse testing conditions. Additionally, the chip formation and development in the planing experiments are qualitatively assessed, particularly in relation to the impact of tool modifications on process forces and temperatures. Each modified tool’s performance is compared against a reference tool without modifications, with percentage differences in forces and temperatures calculated as per Eqs. 7 and 8. The tabular force and temperature values of the respective experiments are available in Table 8.

### Cutting edge characterisation results

**Fig. 11:**
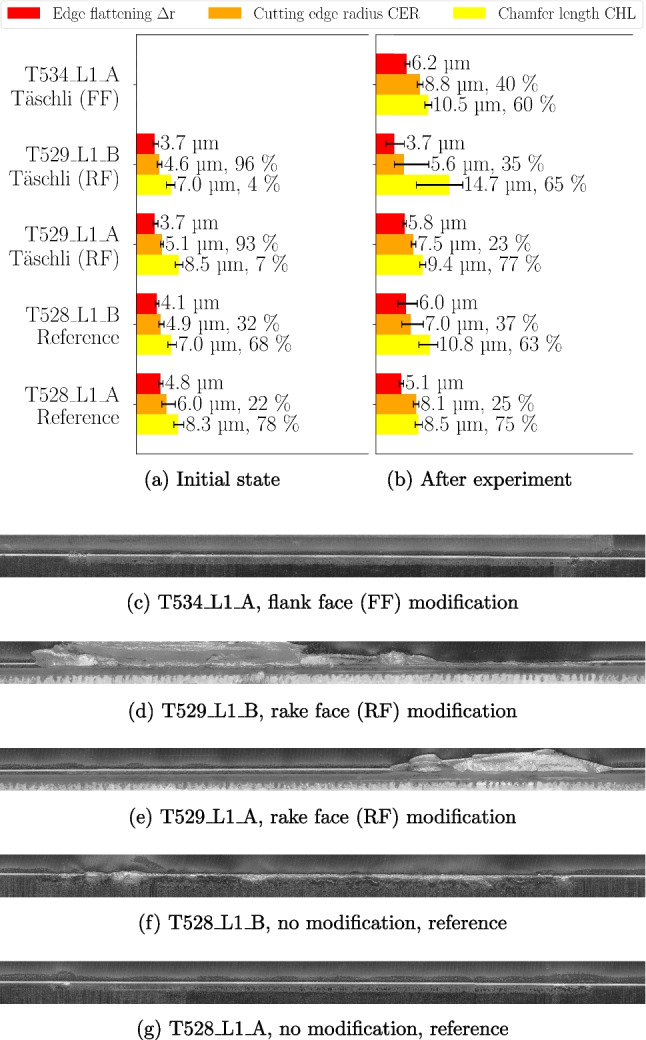
Cutting edge characterisation before and after feed study with a rake angle $\gamma$=20$^{\circ }$ and a clearance angle $\alpha$=7$^{\circ }$ machined at $v_c$=30 m/min for feeds *f*=0.010 to 0.050 mm under dry conditions. The tool designation includes the tool number, followed by the tool side, the cutting edge number, and the position on the respective edge. (**a**) and (**b**) Characterisation of different cutting edges according to Wyen’s algorithm [9] using edge flattening $\Delta r$, cutting edge radius (CER) and chamfer length (CHL). (**c**)–(**g**) Microscopic images of cutting edges after the experiments. Upper half shows the flank and the lower half of the image the rake face, scale of image: 3.0 mm $\times$ 0.2 mm. The cutting length after the experiment for planing is $\approx$ 10 m and for turning $\approx$ 28 m

The 5 cutting edge positions of 3 tools are analysed before and after the experiment in order to ensure that the effect of the tool modification can be differentiated from the deviations of the micro geometry. The cutting length after the experiment for planing, position A, is $\approx$ 10 m and for turning, position B, it is $\approx$ 28 m. The percentage deviations mentioned in the following refer to the min and max values. Before the experiments, $\Delta r$ is between 3.7 and 4.8 µm, CER between 4.9 and 6.0 µm and CHL between 7.0 and 8.5 µm, which is shown in Fig. 11a. The deviations vary from 21 % (CHL) and 30 % ($\Delta r$) respectively, which, in absolute values, equals to deviations <2 µm. The distribution of CER and CHL is inverted between tools T529 and the reference tool T528; T529 exhibits a higher percentage of profiles with CER compared to CHL, whereas T528 shows the opposite. This difference is quantified by percentage values displayed behind the bars, representing the proportion of profiles identified with CER or CHL by the algorithm. The deviations of the CECPs are considered small and the distribution of CER and CHL usually vary at the initial state of the tool before the first use. Consequently, the basis of having comparable starting position for the experiments is given.

At the last experiment, i.e. *f*=0.050 mm, for both planing and turning, severe cutting edge breakouts occurred for T529, which is rake face modified, depicted in Fig. 11 (d) and (e). The break-out of the cutting edge is attributed to the weakening of the tool caused by the modification, which compromised its ability to withstand the tool load. For the characterisation of the cutting edge, the 1.5 mm evaluation length along the cutting edge (see Section 3.4) is chosen at the least damaged area in order to obtain a result that prevailed before the breakout. For T529_L1_B, the results of the CECPs are not considered for the subsequent discussion, since the breakout covers the entire machined area. However, the statements of the neighbouring position, i.e. T529_L1_A, are used to draw conclusions, since their similarities described by the CECPs before the experiment is <0.5 µm. The reference tool T528_L1_B shows also cutting edge breakouts compared to T528_L1_A, which is in accordance with increased CECPs. Generally, after the experiments with *f*=0.050 mm, the CECPs deviate from $\Delta r$ 5.1 to 6.2 µm, from CER 7.0 to 8.8 µm and from CHL 8.5 to 10.8 µm, which is shown in Fig. 11b. The deviations vary from 22 % ($\Delta r$) to 27 % (CHL) respectively and the absolute deviations are within 2.5 µm. Even though the CECPs increased from their initial new state, the changes are considered small and more importantly, the deviations between the different cutting edges are quantifiable similar so that the remaining differences in the micro geometry can only have a minor influence on the process forces and temperatures.

### Planing

**Fig. 12:**
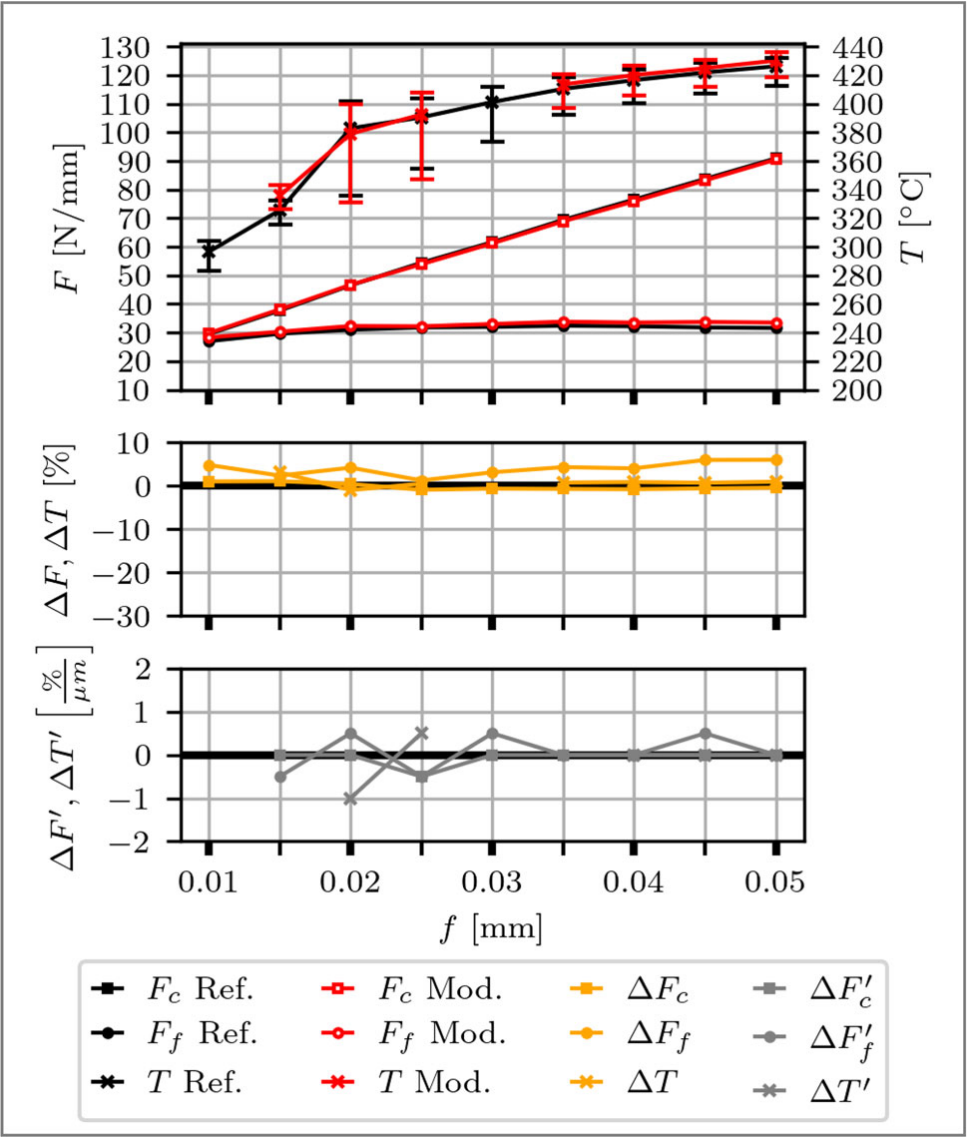
Top: Cutting force $F_c$, feed force $F_f$ and process temperatures *T* over feeds *f* from 0.010 to 0.050 mm of a reference cutting tool (black curves) and a flank face modified tool with a Täschli with a distance to the cutting edge $l_{f1}$ of 33.9 µm and a distance in $F_f$ direction of $\delta _{mod}$ of 4.0 µm (red curves). Middle: Percentage deviations of $\Delta F$ and $\Delta T$. Bottom: Slope of the deviation changes $\Delta F'$ and $\Delta T'$ with respect to the variable *f*

First, a flank face modified tool is evaluated, with the results shown in Fig. 12. At *f*=0.01 mm, the feed is in the order of magnitude of the CECPs and the micro geometry is the dominant factor for the process force generation, which results in $F_c$ and $F_f$ being comparable, i.e. $\approx$ 2 N/mm difference. Across all feeds, $\Delta F_c$ is within 2 %, which is considered small. However, the modified tool exhibits generally larger $F_f$. A maximum deviation of 8 % at *f*=0.045 mm and *f*=0.050 mm is observed. As depicted in Fig. 7b, the CECPs are larger for the modified tool, i.e. 9 % (CER) to 24 % (CHL), which is in accordance with the increased $F_f$.

The deviations of the median in temperature are within 1 % except for *f*=0.015 mm, where the modified tool is 4 % larger. Over the span of different feeds, no effect of the tool modification is identifiable. It is concluded that the $\delta _{mod}$ of 4.0 µm is too large for the given process parameters to show an effect on the process forces or temperatures and consequently, the spring back height is <4.0 µm.

**Fig. 13:**
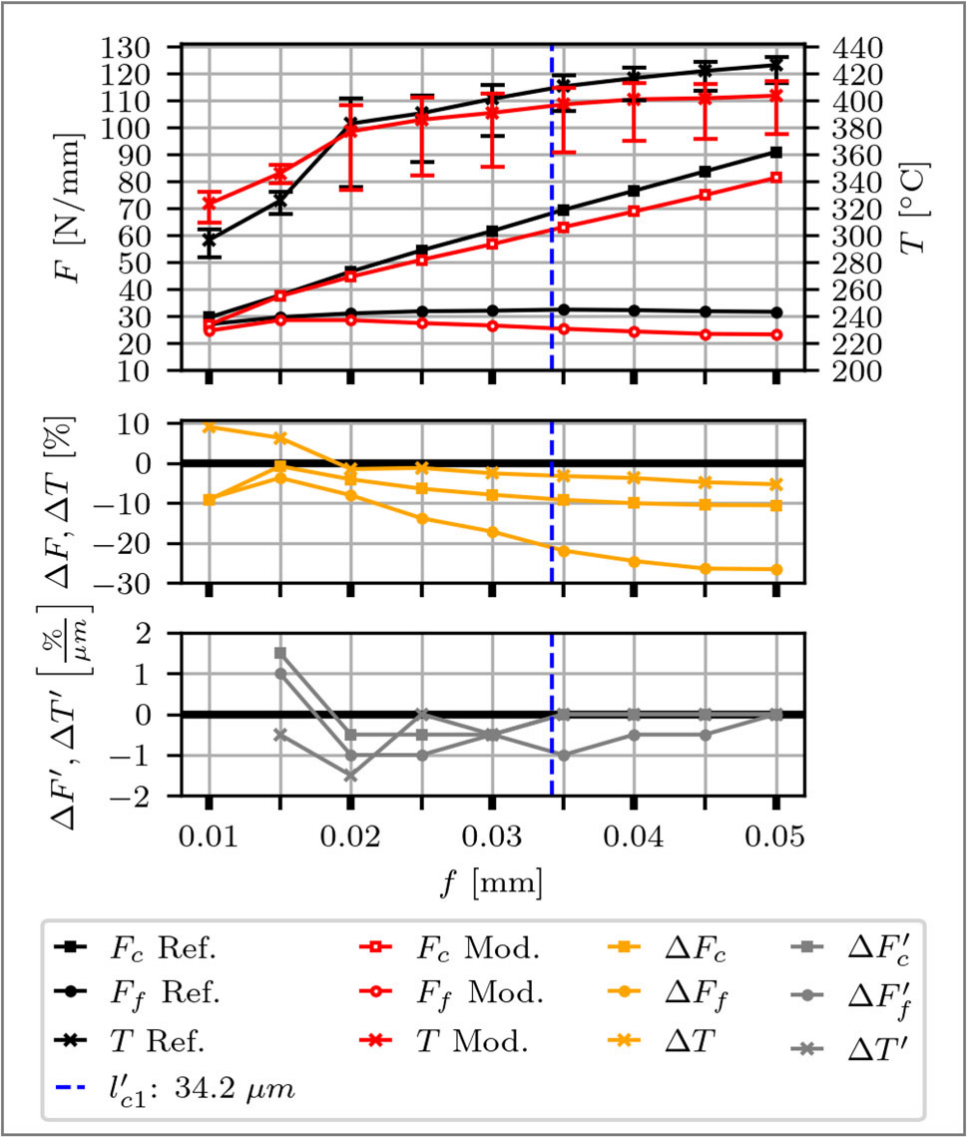
Top: Cutting force $F_c$, feed force $F_f$ and process temperatures *T* of a planing experiment over feeds *f* from 0.010 to 0.050 mm of a reference cutting tool (black curves) and a rake face modified tool with a Täschli with a distance to the cutting edge $l_{c1'}$ of 34.2 µm (red curves). Middle: Percentage deviations of $\Delta F$ and $\Delta T$. Bottom: Slope of the deviation changes $\Delta F'$ and $\Delta T'$ with respect to the variable *f*

Second, a rake face modified tool is discussed, with findings illustrated in Fig. 13. At *f*=0.010 mm, the feed aligns with the magnitude of the CECPs, which associates the cutting edge micro geometry as the significant factor for the process force generation. The modified tool consists compared to a reference tool of a smaller $\Delta r$ (−30 %) and CER (−18 %) and a similar CHL (2 %), correlating with a 9 % reduction in $F_c$ and $F_f$,attributed to these micro geometry differences. At the subsequent *f*=0.015 mm, $\Delta F$ is <5 %, meaning that the effect of the differences in the CECPs diminish. With increasing feed rates ranging from *f*=0.015 to 0.050 mm  the $F_f$ gradually decrease for the modified tool compared to the reference, showing reductions ranging from 4 to 27 %. Meanwhile, the increase in $F_c$ occurs at a slower rate, resulting in differences ranging from 1 to 11 %. The modification is identified to show its first effect between *f*=0.015 and 0.020 µm, evident in $\Delta F_c'$, $\Delta F_f'$ and $\Delta T'$, which means that the chip contact length is restricted by the modification. The $\Delta F_c'$ decrease with increasing *f* and remains within <0.1 %/µm after *f*=0.020 mm, marking a transition point at *f*=0.025 mm for small changes. Similarly, $\Delta F_f'$ decrease with increasing *f* with a transition point at *f*=0.040 mm, where the change remains <0.1 %/µm, which coincides with the distance to the cutting edge $l_{c1'}$ of 34.2 µm. Consequently, part of the feed, and therefore the chip itself, may begin to penetrate into the modification, diminishing the impact of the restricted contact length. The temperature follows the curve of $F_c$, also visible for $\Delta F_c$ and $\Delta T$. At smaller feeds, i.e. *f*=0.010 mm and *f*=0.015 mm, the median temperature of the modified tool is larger by 4 to 6 %, consistent with the observations for the flank face. From *f*=0.020 to 0.050 mm, a continuous decrease of the median temperature of the modified tool is observed, reaching −5 % for $\Delta T$. Error bars indicate, right-skewed temperature distributions for the modified tool, concentrated towards lower Q1 values, with a span from Q1 to Q3 up to twice larger. A comparison of Q1 values reveals temperatures up to 9 % lower for the modified tool.

Overall, the results demonstrate that rake face modified tools exhibit promising potential for reducing process forces and temperatures as soon as the chip contact length is reduced by the modification. However, as *f* approaches $l_{c1'}$, where a part of the feed has the possibility to enter the modification, reductions in process forces and temperatures stabilise, indicating a plateau effect. Therefore, the ideal feed rate is derived between 0.040 and 0.045 mm, taking into consideration that cutting edge breakout occurred at *f*=0.050 mm.

For analysing the chip formation, the initial chip segment, i.e. $\approx$ 0.2 mm, is examined under a microscope at 1500X magnification, specifically for feeds ranging from 20 to 50 µm, where the chips are embedded and etched with TitanEtch to visualise the microstructure. Despite some variations in the chip formation over the 200 mm stroke length and across repeated experiments, the initial chip segment is observed to be representative of the overall chip formation process. This is because the initial segment is formed under stable orthogonal cutting conditions, which typically reflect the trends observed throughout the entire stroke. Additionally, given the short cutting time of approximately 0.4 s at a constant cutting speed of 30 m/min, temperature effects and other variations are minimised during a single stroke, supporting the consistency of the initial segment’s characteristics.

For the first initial feeds, i.e. 20 to 30 µm, no significant difference is observed for the chip morphology. For the subsequent feeds, where *f* becomes larger than $l_{c1'}$, the chips produced by the reference tool generally appear rougher and less uniform compared to those produced by the modified tool. As the feed increases, the chips from the reference tool show more pronounced jagged edges, typical for chip segmentation, and variations in texture, suggesting possible variations in cutting efficiency. The modified tool consistently produces smoother, more uniform chips, which is associated with the better performance in terms of lower process forces and temperatures shown in Fig. 13. The visual comparison underlines that the modified tool is superior in producing more controlled and consistent chips, which is crucial for maintaining quality and efficiency in machining operations. Notably, the chip of the modified tool at *f*=0.050 mm shows after the first contact point with the tool, an impression in the shape of the tool modification, which confirms that the part of the feed above $l_{c1'}$ enters the modification and therefore alters the chip flow.

**Fig. 14:**
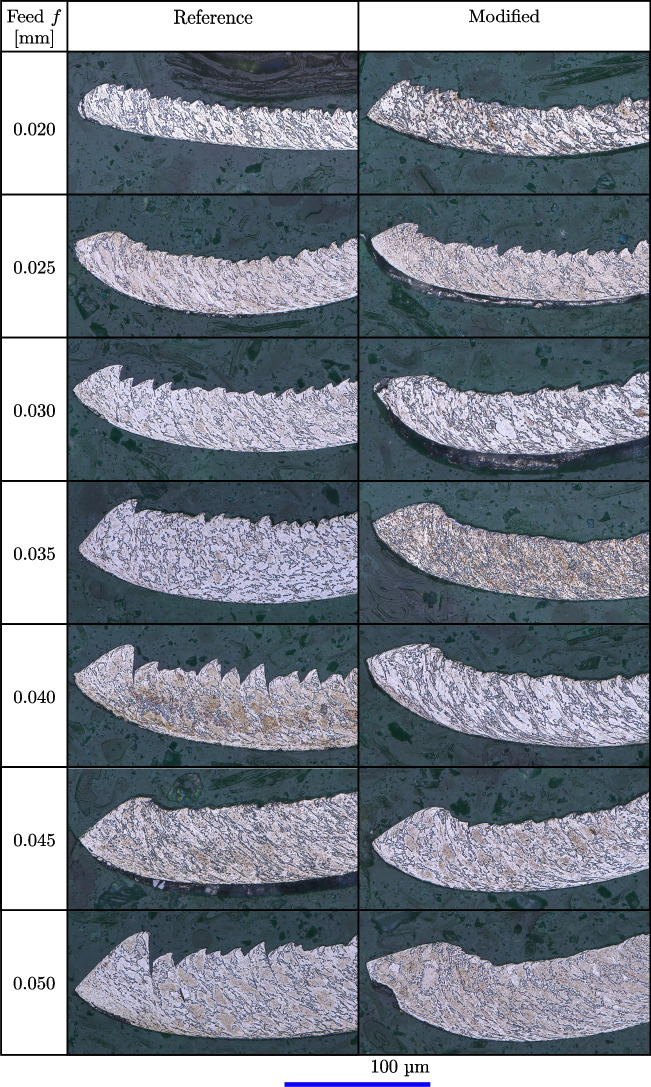
Qualitative comparison of the chip formation of the initial chip segment, i.e. $\approx$ 0.2 mm, of a reference cutting tool and a rake face modified tool with a Täschli with a distance to the cutting edge $l_{c1'}$ of 34.2 µm for feeds ranging from *f*=0.020 to 0.050 mm µm

**Fig. 15:**
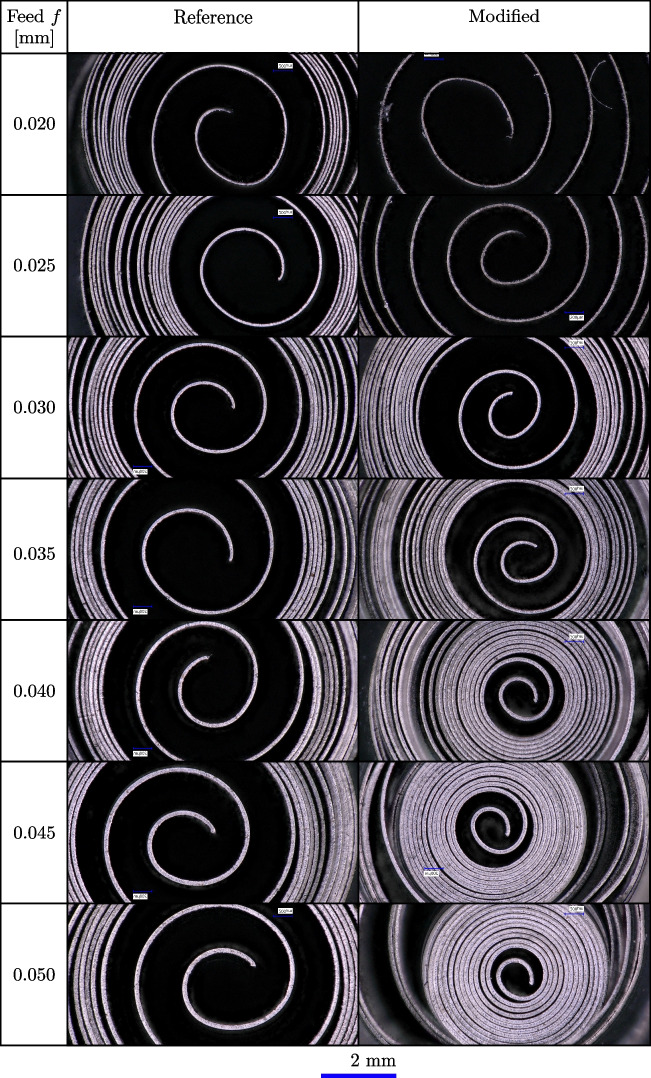
Qualitative comparison of the chip formation and development of a reference cutting tool and a rake face modified tool with a Täschli with a distance to the cutting edge $l_{c1'}$ of 34.2 µm for feeds ranging from *f*=0.020 to 0.050 mm

The chip formation as presented in Fig. 14 influences the subsequent chip development as visualised in Fig. 15, which is discussed in the following. The chips formed by the reference tool show a consistent spiral shape across all feeds, which implies a relatively consistent material deformation and removal process. The chips from the modified tool exhibit variations in the spiral formations. At lower feeds, i.e. *f*=0.020 to 0.025 mm, the center of the chips has similar chip curling radii as the reference tool. For feeds where *f* exceeds $l_{c1'}$, the modified tool produces increasingly tight spirals, resulting in reduced chip curling radii. This indicates that the modification affects the material flow.

**Fig. 16:**
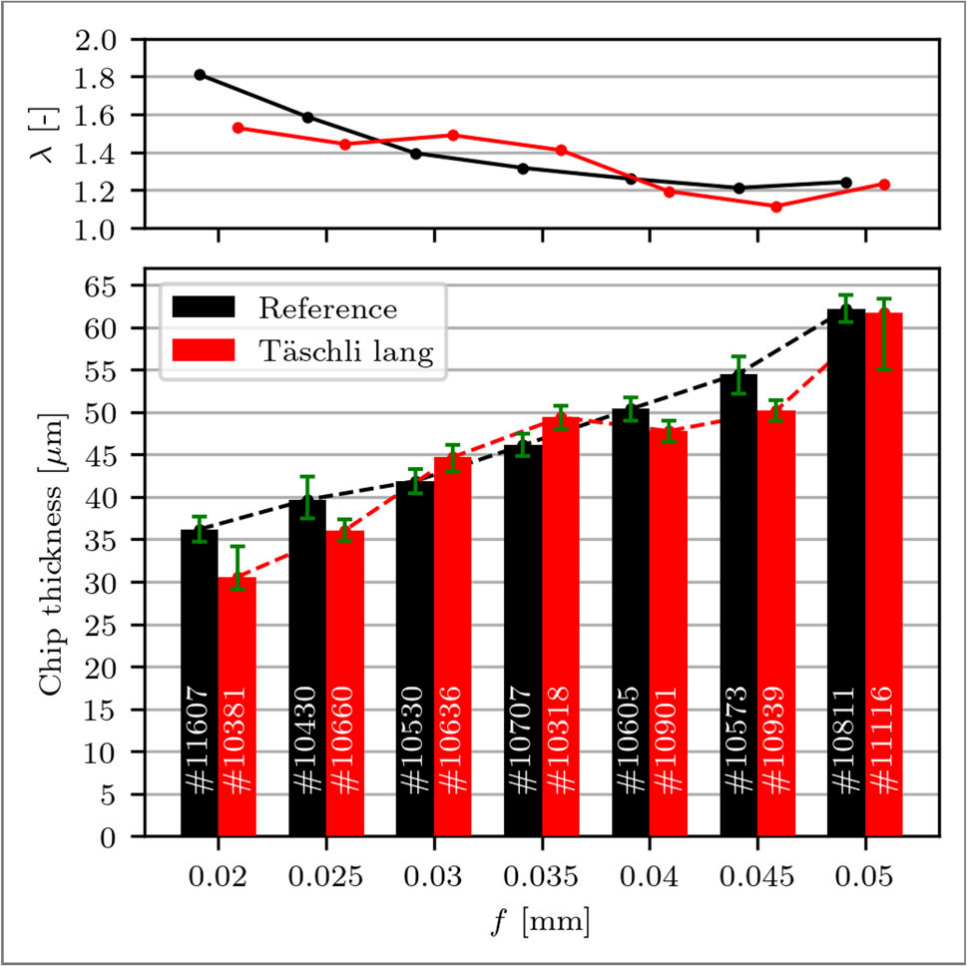
Plot with chip compression ratio $\lambda$ (top) and bar plot with the chips thickness (bottom) over feeds *f*=0.020 to 0.050 mm of a reference cutting tool (black) and a rake face modified tool with a Täschli with a distance to the cutting edge $l_{c1'}$ of 34.2 µm (red). The number of measurements # for each thickness is indicated in white in the respective bars

Finally, the chip resulting from one stroke can be analysed at different locations to evaluate the chip thickness. It is revealed that for small changes in the process forces, i.e. at *f*=0.020 to 0.025 mm, the chip thickness of the modified tool is 6 to 13 % smaller, depicted in Fig. 16. When *f* approaches $l_{c1'}$ at *f*=0.030 to 0.035 mm, the chip thickness of the modified tool is 7 % larger than the reference tool. The restricted contact length in combination with the reduced cutting forces may result in less deformation of the workpiece material and less compression at the cutting zone, potentially leading to thicker chips. However, when *f* exceeds $l_{c1'}$ at *f*=0.040 to 0.050 mm, the chip thickness of the modified tool is again smaller despite the lower process forces. This suggests that the chip formation in the shear zones is changed so that less work is required to form the chip, which is in agreement with the observations depicted in Fig. 14. It should be noted that the chip thickness shows naturally occurring deviations, thus, at least 10’000 measurement points resulting in $\approx$ 2.5 mm chip length per chip are considered for the evaluation, indicated in white font in the respective bars of Fig. 16. The same trend is observed with the chip compression ratio $\lambda$, as shown in Fig. 16.

### Turning

Turning experiments are exclusively performed using a rake face-modified tool. This decision stems from the observation that the flank face modified tool, as detailed in Section 4.2, did not yield any significant effects on the process forces and temperatures. Moreover, temperature measurements are unfeasible due to inconsistent signal intensity, likely stemming from chip-fiber contacts on the rake face. During planing, the chip length is limited by the length of the workpiece, i.e. 0.2 m, enabling the formation of a chip curl. In contrast, during turning, the chip length per experiment is primarily equal to the cutting length of $\approx$ 3 m, which develops along the rake face and thus inhibits the formation of an orthogonal chip curl. Consequently, this prevents the undisturbed process temperature measurement with the fibre positioned orthogonal to the to the cutting edge, as shown in Fig. 9.

**Fig. 17:**
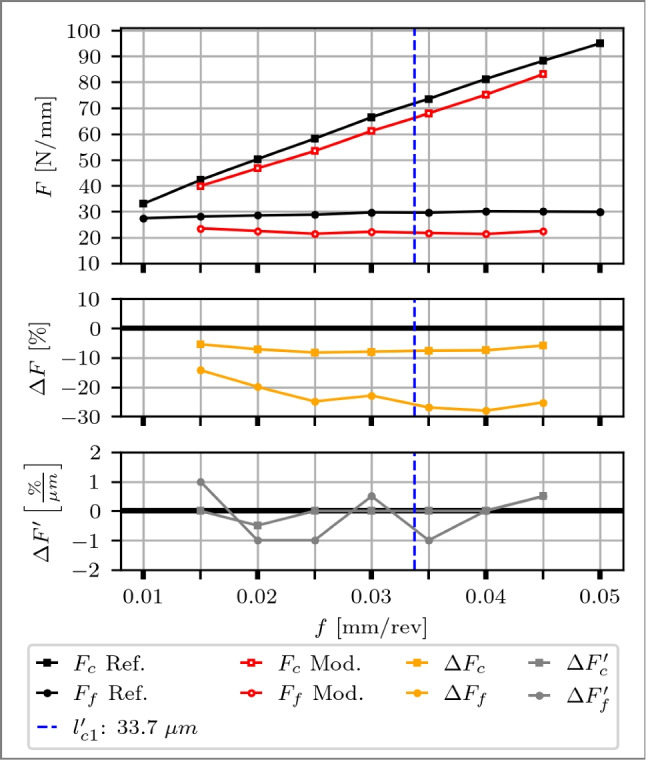
Top: Cutting force $F_c$, feed force $F_f$ from a turning experiment over feeds *f* from 0.010 to 0.050 mm/rev of a reference cutting tool (black curves) and a rake face modified tool with a Täschli with a distance to the cutting edge $l_{c1'}$ of 33.7 µm (red curves). Middle: Percentage deviations of $\Delta F$. Bottom: Slope of the deviation changes $\Delta F'$ with respect to the variable *f*

For *f*=0.01 mm/rev, no constant steady-state force could be determined for the modified tool attributed to the short cutting length of $\approx$ 3 m resulting in 5 rotations, which was insufficient to overcome the initial run-in behaviour. Subsequent tests at *f*=0.015 mm/rev, revealed reductions of −5 % and −17 % for $F_c$ and $F_f$ respectively, as depicted in Fig. 17. Despite comparable CECPs before and after experiments, as detailed in Section 4.1, which minimises the effect of the cutting edge geometry on the process forces, the modified tool shows more radii, i.e. 96 %, and the reference tool consists of more chamfers, i.e. 78 %, which can partially be associated with the lower force of the modified tool. However, the primary force reduction is linked to the restricted chip contact length. Across increasing feed rates from *f*=0.015 to 0.040 mm/rev, the process forces tend to decrease for the modified tool compared to the reference tool. $F_c$ reduces from 6 to 8 % and $F_f$ from 16 to 27 %. Notably, $\Delta F'_c$ remains <0.1 %/µm over the entire feed range, while $\Delta F'_f$ decrease with increasing *f*, with a transition point at *f*=0.030 mm/rev, remaining <0.1 %/µm. When *f* exceeds $l_{c1'}$ between *f*=0.030 mm/rev and *f*=0.035 mm/rev, where a part of the feed of the subsequent chip can begin to penetrate into the modification, a slight simultaneous change for $F_c$ and $F_f$ is present, especially evident for $\Delta F'$. At *f*=0.050 mm/rev, a meaningful force evaluation is not possible due to cutting edge breakouts, rendering the results inconclusive.

**Fig. 18:**
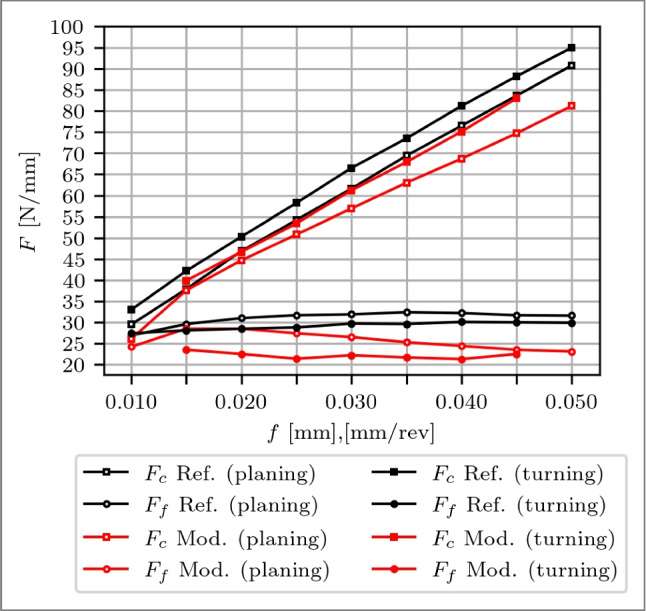
Cutting force $F_c$ and feed force $F_f$ over feeds *f* from 0.010 to 0.050 mm/rev of a reference cutting tool for planing (black markers with white filling) and turning (black markers) and a rake face modified tool with a Täschli with a distance to the cutting edge $l_{c1'}$ of 33.7 µm (red markers with white filling) for planing and $l_{c1'}$ of 34.2 µm (red markers) for turning

In conclusion, the rake face-modified tool exhibited process force reductions due to the restricted chip contact length. As *f* approaches $l_{c1'}$, allowing a part of the feed of the subsequent chip to penetrate in the modification, reductions in process forces stabilise, signifying a plateau effect. The ideal feed rate is derived between 0.035 and 0.045 µm/rev, considering that cutting edge breakout occurred at *f*=0.050 mm/rev.

### Planing vs. turning

Comparing the reference process forces in Fig. 18, $F_c$ of the turning experiments exceeds that of planing by 4 to 5 N/mm, while $F_f$ is generally smaller, from 1 to 3 N/mm. This disparity is attributed to the different process kinematics and material batches. Despite these variances, both processes exhibit comparable trends. Notably, with the modified tools, $F_f$ achieved a maximum reduction of 27 %, wheras $F_c$ exhibited a variation range from 8 % for turning to 11 % for planing. The modification’s impact initiated earlier and more significantly during turning, i.e. at *f*=0.015 mm, compared to planing, i.e. at *f*=0.020 mm, implying a longer chip contact length for turning. Furthermore, while $F_c$ and $F_f$ gradually decreased over the feed range in planing experiments until a plateau point at *f*=0.040 mm, turning experiments revealed a stabilising point as early as *f*=0.030 mm.

**Fig. 19:**
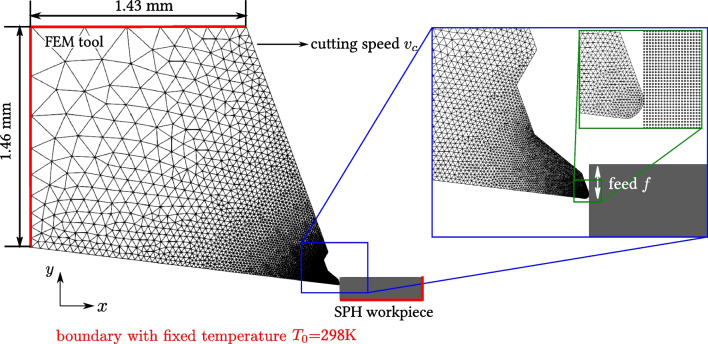
Schematic of hybrid SPH-FEM simulation model with a rake face modified tool with a Täschli with a distance to the cutting edge $l_{c1'}$ of 34.2 µm

**Table 5:** Result of inversely identified Johnson-Cook material parameters comparing simulated and experimental forces, where the $\Delta$ is expressed as $F^{sim}-F^{\exp }$

$F_c^{\exp }$	$\Delta F_c$	$\Delta F_c / F_c^{\exp }$	$F_f^{\exp }$	$\Delta F_f$	$\Delta F_f / F_f^{\exp }$
[N/mm]	[N/mm]	[%]	[N/mm]	[N/mm]	[%]
98.3	0.1	0.1	39.7	0.03	0.1

## Simulation

This section moves from experimental investigations to simulation analyses to deepen the understanding of the experimental planing results presented in Section 4.2. Initially, the modeling of the orthogonal cutting process in numerical simulations is described. Subsequently, the simulation results are presented, offering additional insights and complementing the experimental findings through comparative evaluations of chip formation and development. Finally, the section concludes with an assessment of the accuracy and relevance of the simulations in replicating experimental outcomes, specifically focusing on the chip compression ratio $\lambda$ and process forces.

### Modeling of orthogonal cutting simulations

The orthogonal 2D cutting simulations are performed using the software iwf_mfree developed at the Institute of Machien Tools and Manufacturing (IWF) of ETH ZÜRICH. The configuration of the cutting simulation is depicted in Fig. 19.

The Ti6Al4V workpiece is discretised into SPH particles to better capture large deformations and facilitate thermomechanical analysis. In this study, the particle spacing is set to 1 µm. The uncoated cemented carbide tool is modeled as a rigid body using a linear triangular finite element mesh solely for transient thermal analysis. The assumption that the tool behaves as a rigid body is justified by the fact that the deformation of the tool is negligible compared to the large plastic deformations occurring in the workpiece material, particularly in the chip formation zone. This is especially true for the cemented carbide with 6 % cobalt content used in this study, which is known for its high hardness and brittleness. The mesh size varies, with the smallest dimension around 1 µm at the cutting edge. The tool geometry of the modified tool is modeled based on the tool measurements of tool T529_L1_A from Table 4 and Fig. 11b, where a cutting edge radius of 7.5 µm is used. The simulation model is optimised for Graphics Processing Unit (GPU) acceleration, ensuring efficient computations. For detailed information on model assumptions, discretisation of governing equations, contact algorithms, and GPU implementation, readers are referred to the original works [2, 19, 20].

The material model in iwf_mfree used to simulate the plastic deformation is the flow stress model according to Johnson and Cook (JC) [21]. The law used to describe the isotropic hardening of the workpiece is given in:

9 $$\begin{aligned} {\begin{matrix} \sigma _Y = & (A + B \cdot \varepsilon _{pl}^n) \cdot \\ & \left( 1+C \cdot \ln \frac{\dot{\varepsilon }_{pl}}{\dot{\varepsilon }_{pl}^0} \right) \cdot \left( 1-\frac{T-T_{ref}}{T_f-T_{ref}} \right) ^m \end{matrix}} \end{aligned}$$

The parameters *A*, *B*, *C*, *m* and *n* are the material parameters, $\varepsilon _{pl}^n$ denoting the current plastic strain, $\dot{\varepsilon }_{pl}$ indicating the current plastic strain rate, *T* signifying the current temperature, $T_f$ the melting temperature, $T_{ref}$ the reference temperature and $\dot{\varepsilon }_{pl}^0$ represents the reference plastic strain rate. The first two terms account for hardening resulting from plastic strain and plastic strain rate, while the third term governs thermal softening with increasing temperature.

In the study by [22], twenty distinct sets of Johnson-Cook parameters for Ti6Al4V are examined and compared. The analysis reveals significant variations among these parameter sets, underscoring the profound impact that the selection of Johnson-Cook parameters can have on the accuracy of flow stress predictions and, consequently, on the outcomes of machining modeling. An inverse parameter identification, as outlined by [10], is conducted to refine the material parameters *A*, *B*, *C*, *m*, and *n*. This method employs a orthogonal cutting experiment to optimise material parameters within SPH simulations, aiming to reduce the discrepancies between the simulated and experimentally measured process forces. The cutting experiment employs a sintered, uncoated, and ground cutting insert with the tool geometry $\gamma$=10$^{\circ }$, $\alpha$=7$^{\circ }$, and $r_n$=5 µm, similar to the tools used in this study. The process conditions are $v_c$=30 $\text {m}/{\min }$ and *f*=0.050 mm. However, due to insufficient resolution when scanning the cutting edge with the confocal microscope, $r_n$ is initially measured as 17 µm instead of 5 µm. Nevertheless, the obtained parameters predict the behaviour of the process forces well, as detailed in the upcoming Section 5.3. The inverse parameter identification optimisation process involves 1118 iterations, requiring $\approx$ 10 weeks of computational time, where the best-identified parameter set results in 0.1 % discrepancy in the cutting and feed force compared to the experiment, illustrated in Table 5. In this context, $\Delta F_c$ and $\Delta F_f$ are $F^{sim}-F^{\exp }$.

**Table 6:** Material properties of Ti6Al4V used for the cutting simulations

Parameter	Symbol	Value	Unit	Data source / derived from
Young’s modulus	*E*	130	GPa	Table 2
Poisson ratio	$\nu$	0.35	−	Table 2
Density	$\rho$	4430	kg/m$^{3}$	Table 2
Specific heat	$c_p$	526	J/kg K	[23]
Thermal conductivity	$\lambda$	6.8	W/m K	[23]
Friction coefficient	$\mu$	0.35	−	[8]
Taylor-Quinny coefficient	$\eta$	90	%	[24]
JC constant	*A*	1084.6	MPa	
JC constant	*B*	843.0	MPa	
JC constant	*C*	0.0296	−	
JC constant	*m*	0.5667	−	
JC constant	*n*	0.1815	−	
JC constant	$\dot{\varepsilon }_{pl}$	1.0	1/s	
Reference temperature	$T_{ref}$	300	K	
Melting temperature	$T_f$	1836	K	

All the material properties used for the subsequent simulations are listed in Table 6. The heat conduction coefficient at the tool-chip and tool-workpiece interfaces are set as $5\cdot 10^6$ W/(m$^2$K), and the proportion of the frictional heat partitioned to the tool is 0.5.

### Chip formation and development

The simulations aim to enhance the experimental findings by providing a detailed qualitative analysis of chip formation and development. This includes evaluating the equivalent accumulated plastic strain $\bar{\varepsilon _p}$, analysing the temperature distribution, and predicting the chip contact length $l_c$.

**Fig. 20:**
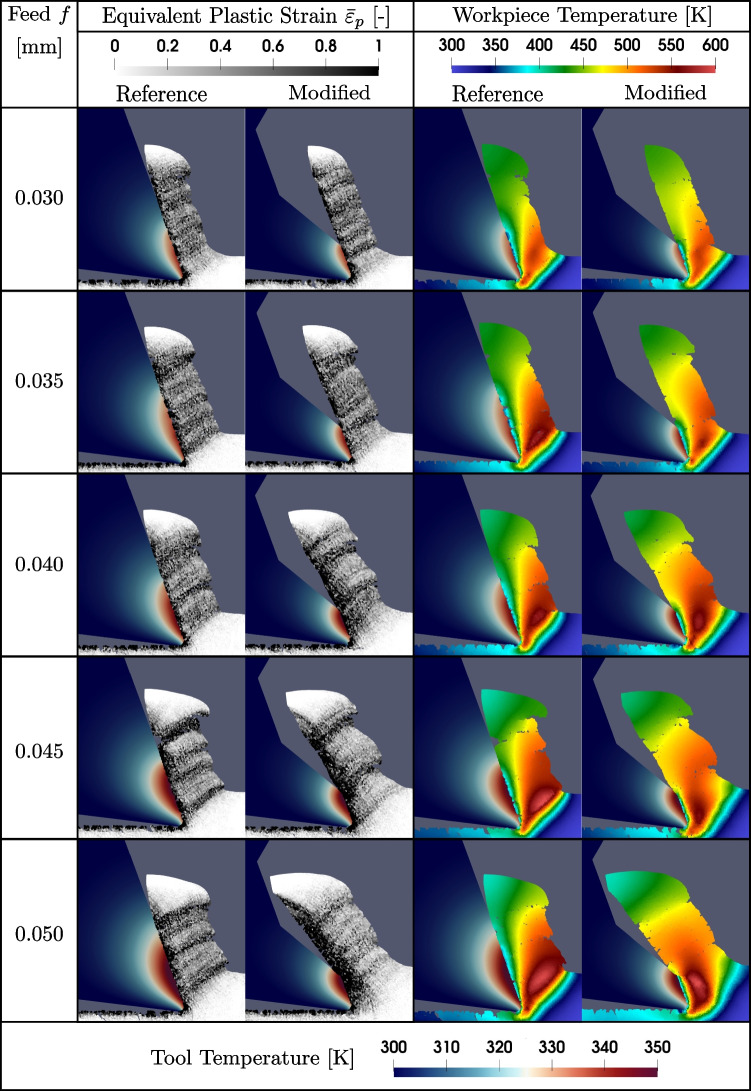
Visualisation of the chip formation from SPH simulation results showing the tool and chip temperature and the equivalent accumulated plastic strain of a reference cutting tool and a rake face modified tool with a Täschli with a distance to the cutting edge $l_{c1'}$ of 34.2 µm over feeds *f* from 0.030 to 0.050 mm. For representation purposes, an equivalent accumulated plastic strain limit of 1 is used. The range of the tool temperature is chosen as 300 to 350 K and for the chip 300 to 600 K

The chip formation is shown for *f*=0.030 to 0.050 mm in Fig. 20, characterised by $\bar{\varepsilon _p}$, the tool and the workpiece temperature, analysed at the beginning of the cutting process for an approximate cutting length of 0.2 mm. First, the $\bar{\varepsilon _p}$ is discussed, where the limit of 1 is chosen for representation purposes. At feeds of 0.030 mm and 0.035 mm, there is no significant difference in chip formation between the modified and unmodified tools. However, at 0.040 mm, the chip from the modified tool begins to bend inside the tool modification, as *f* exceeds $l_{c1'}$. With increasing feed, the thickness of the sheared layer at the chip’s contact side with the rake face enlarges due to the tool modification, altering the chip flow direction. The modification’s inlet angle, $\gamma _2$, acting as a secondary rake angle, begins to influence the cutting process more prominently. This modification-induced dynamic persists at higher feeds. At *f*=0.050 mm, the reference tool displays shear bands across the chip’s thickness, a feature absent in the modified tool, which instead exhibits an increase in plastic strain at the chip face. Notably, the tool modification tends to reduce chip segmentation, particularly at feeds exceeding 0.040 mm, compared to the reference tool. This leads to more continuous chip formation, despite the sheared layer at the chip’s contact side constituting nearly 25 % of the total chip thickness. The changes in chip morphology, validated by experimental findings shown in Fig. 14, can be attributed to two primary factors: the redirection of chip flow by the modification, altering the effective shear angle, and a reduction in contact length, which decreases friction and thus chip flow resistance. These results validate the simulation’s ability to replicate experimental outcomes, enhancing the understanding of the machining process.

Chip formation can also be assessed in terms of temperature development and distribution, both in the tool and chip, as illustrated in Fig. 20, even though the temperature in the tool only reflects its development during the initial stage of the cutting process. A tool temperature range of 300 to 350 K and for the chip 300 to 600 K is selected in the graphical display. Generally, the temperature influenced zone on the rake face is smaller for the modified tool due to the restricted contact length and therefore less heat is conducted into the tool. Furthermore, the larger *f*, the more heat is generated, which corresponds to both, the tool and chip temperature. At *f*=0.030 mm, no significant difference in the chip temperatures is observed. For *f*=0.035 mm, the chip of the modified tool exhibits smaller overall temperatures, especially looking at the primary shear zone. This effect is not continued for the subsequent *f*=0.040 mm, where *f* becomes larger than $l_{c1'}$, which marks the beginning of a change in the chip flow. Continuing increasing *f* to 0.045 mm shows again a less distinct temperature development in the primary shear zone for the modified tool as for *f*=0.035 mm, which is explained by the more dominant change of the chip flow around the tool modification and in agreement with the observations of $\bar{\varepsilon _p}$. This trend continues for *f*=0.050 mm, where the temperature of the modified tool in the primary shear zone close to the free surface is $\approx$ 20 % lower. Consequently, the modification at larger feeds is associated to alter the stress distributions, indicated by the plastic strain, and heat generation during cutting, which influences how the workpiece material deforms and subsequently shears.

**Fig. 21:**
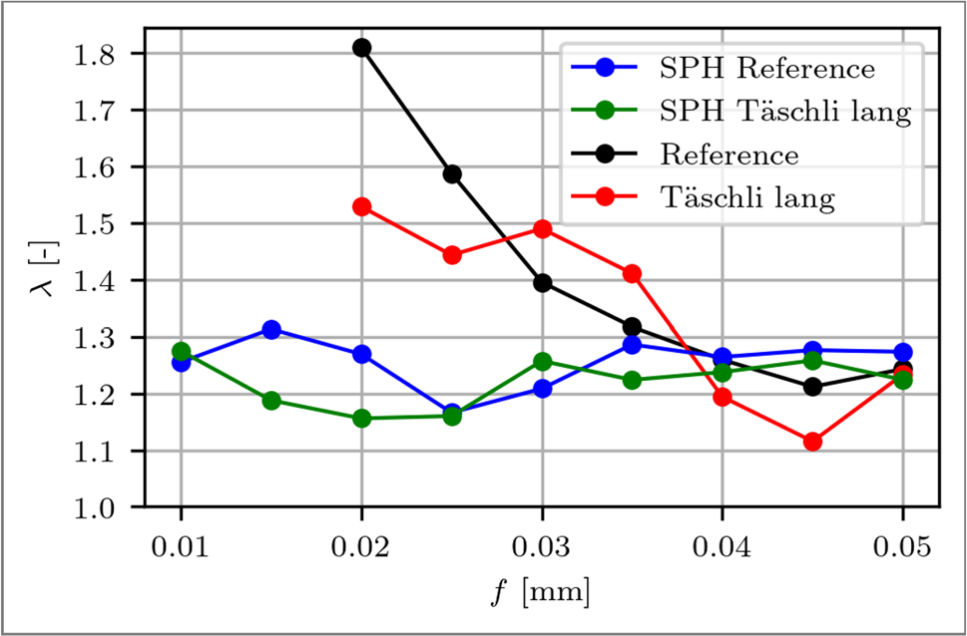
Comparison between experimental and SPH simulation results of the chip compression ratio $\lambda$ over feeds *f*=0.020 to 0.050 mm µm of a reference cutting tool (experiment: black, SPH: blue) and a rake face modified tool with a Täschli with a distance to the cutting edge $l_{c1'}$ of 34.2 µm (experiment: red, SPH: green)

In terms of the compression ratio $\lambda$, the simulation shows less variation compared to experimental results across the entire feed range, as depicted in Fig. 21. Specifically, within the feed range from 0.020 to 0.035 mm, the simulation underestimates $\lambda$, and thus the chip thickness, though it gradually aligns more closely with experimental observations. Notably, $\lambda$ for the modified tool is higher than that of the reference tool as the feed approaches the onset of the modification at *f*=0.03 mm. Upon reaching the start of the modification, i.e. $l_{c1'}$=34.2 µm, the simulation shows a decrease in $\lambda$, diverging from the experimental trend, which continues to show a higher $\lambda$. For the subsequent feeds, $\lambda$ of the modified tool remains for the simulation and for the experiment below the one of the reference.

**Fig. 22:**
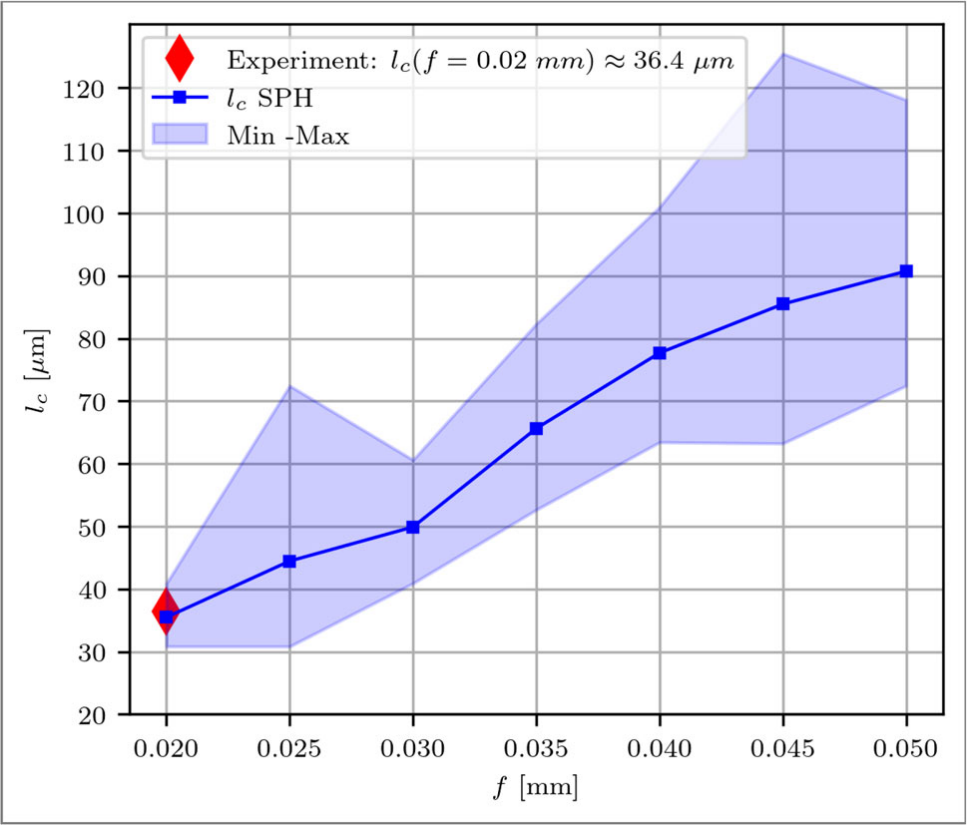
Chip contact length $l_c$ over feeds *f*=0.020 to 0.050 mm of a reference cutting tool. The red diamond-shape marker is the experimental validation point

The predicted $l_c$ from the simulation can be validated at a specific point using the modified tool, where at *f*=0.02 mm the contact length is $l_c$ $\approx$ 36.4 µm determined through the experimental force evaluation from Fig. 13. The contact length is calculated by identifying the furthest point from the cutting edge where the chip contacts the rake face. The maximum recorded value, Max, represents the farthest contact point, while the minimum value, Min, represents the closest contact point to the cutting edge. The evaluation of $l_c$ over *f* is illustrated in Fig. 22. Occasional uneven distribution of particles introduces some uncertainties when considering the chip contact length, which is shown by the Min and Max values. At *f*=0.02 mm, the averaged simulation result and the experimental value match <4 %, where the simulation predicts $l_c$=37.8 µm. This accuracy is supported by the consistent prediction of the cutting force $F_c$ within a single-digit percentage error range, as discussed in Section 5.3.

### Process forces

**Fig. 23:**
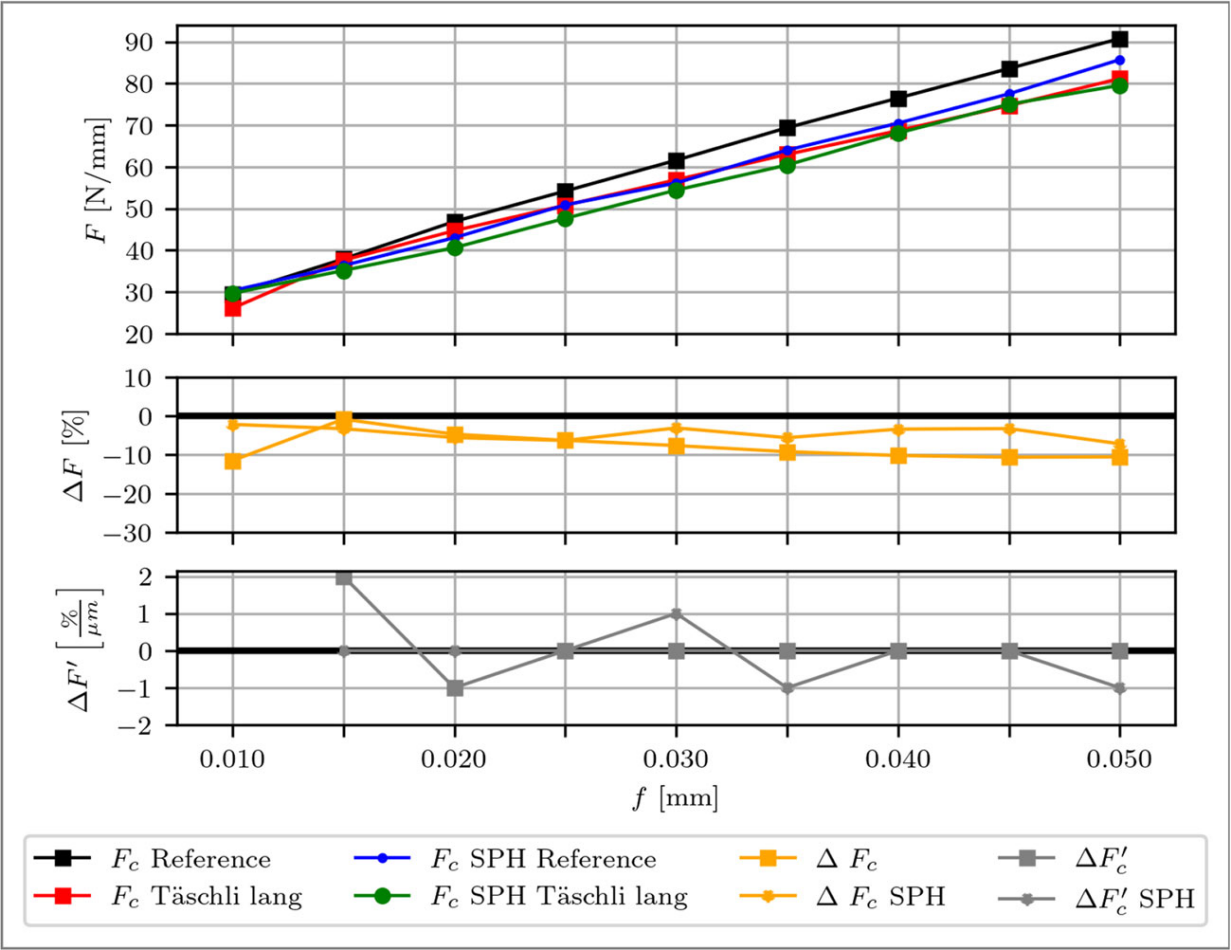
Comparison between experimental and SPH simulation result of the cutting force $F_c$ over feeds *f* from 0.010 to 0.050 mm. Top: Cutting force $F_c$ of a reference cutting tool (experiment: black, SPH blue) and a rake face modified tool with a Täschli with a distance to the cutting edge $l_{c1'}$ of 34.2 µm (experiment: red, SPH: green). Middle: Percentage deviations of $\Delta F$. Bottom: Slope of the deviation changes $\Delta F'$ with respect to the variable *f*

The SPH simulation effectively captures $F_c$ for the reference case, as illustrated in Fig. 23. Excluding the feed of *f*=0.010 mm, the simulation achieves a mean error of 6.1 %, with the simulated force being underestimated. The modified tool demonstrates even greater accuracy, achieving a mean error of 2.6 % without considering the initial feed, while there are instances of both underestimation and overestimation. This relatively accurate prediction of the cutting force with the modified tool suggests that the numerical simulation can effectively reflect the chip formation behavior in the primary deformation zone. This is under the condition that uncertainties caused by the dynamics of chip sliding and excessively long contact lengths are excluded. Since the simulation results of the reference tool are underestimated, $\Delta F_c$ is less pronounced for the simulation results, however, the trend is predicted correctly. $\Delta F'_c$ reveals that the simulation results fluctuate within ±1 %/µm over the entire feed range.

**Fig. 24:**
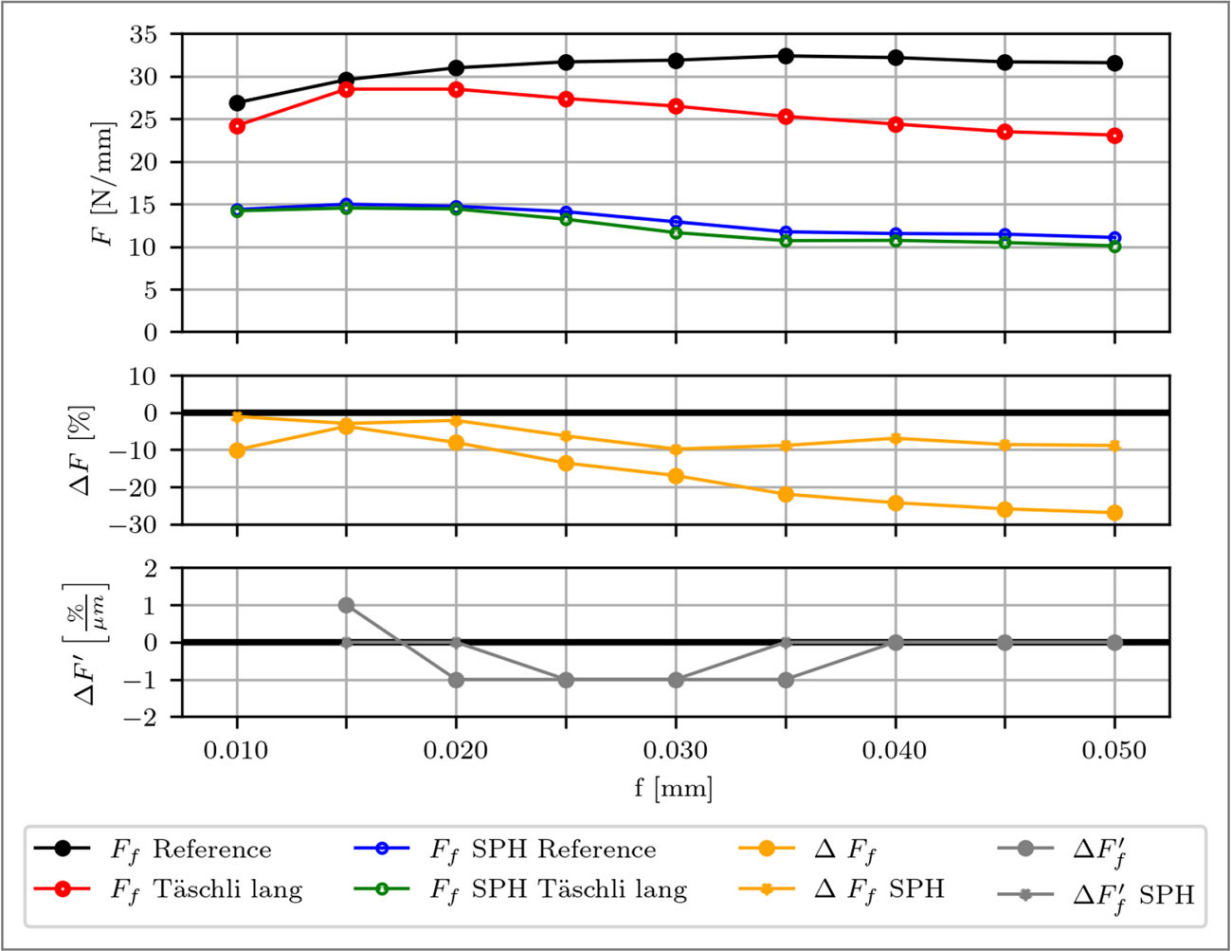
Comparison between experimental and SPH simulation result of the feed force $F_f$ over feeds *f* from 0.010 to 0.050 mm. Top: Feed force $F_f$ of a reference cutting tool (experiment: black, SPH blue) and a rake face modified tool with a Täschli (Swiss German for small pocket) with a distance to the cutting edge $l_{c1'}$ of 34.2 µm (experiment: red, SPH: green). Middle: Percentage deviations of $\Delta F$. Bottom: Slope of the deviation changes $\Delta F'$ with respect to the variable *f*

In the simulation, $F_f$ is approximately underestimated by 50 %, as depicted in Fig. 24. Notably, while the reference tool exhibits an approximately constant force behavior after *f*=0.015 mm, the simulation shows a decrease in $F_f$ with increasing feed, mirroring the behavior of the modified tool. This trend closely aligns with experimental results using the modified tool but fails to capture the trend for the reference tool, indicating that the simulation may not fully account for additional tool-chip contact areas observed in experiments due to kinematic effects like chip waving or vibrating. It is important to note that accurately modeling the feed force is inherently complex due to its high sensitivity to a multitude of factors, which are extensively discussed in [2] and accurately capturing all these variables simultaneously in a simulation is extremely challenging, which goes beyond the scope of this work. Moreover, the underestimation of the feed force is a known issue in simulations involving sharp tools, as for example elaborated in [2] and [25].

Despite these discrepancies, both the experimental and simulation results identify a transition point at *f*=0.02 mm where the modification begins impacting the process forces. As feed increases, the simulation stabilises at *f*=0.035 mm, and the experiment at *f*=0.040 mm, as indicated by changes in $\Delta F'$. The simulation reaches a plateau effect at the beginning of the modification at $l_{c1'}$=34.2 µm, before a part of the feed of the subsequent chip can penetrate into the modification. In contrast, the experiment continues to show a decrease in $F_f$ by −1 %/µm. These observations underscore the effectiveness of the modified tool in reducing process forces and highlight the potential discrepancies between simulated and real-world behaviors due to unmodeled physical interactions. The nearly stable discrepancies between the simulated and the experimental feed forces using the modified tool indicate the presence of systematic error sources. These errors may stem from factors such as the limitations of representing the cutting edge-machined surface contact in 2D modeling, among others.

## Conclusion

This paper details the progress in dry orthogonal machining of Ti6Al4V using cutting tools with ultra-short pulsed laser-ablated tool geometry modifications. The study analyses the effect of modifying the rake and flank faces of cutting inserts to optimise the machining process. The key highlights of this work include:

1. Laser modification of tools: The paper outlines a method for creating precise modifications on cutting tools, which allows for adjustments to the rake and flank faces with micrometer accuracy. These modifications are aimed at minimising process forces and temperatures.
2. Experimental setup and results: This study conducts extensive experimental evaluations at varying feeds from *f*=0.010 to 0.050 mm to assess the effectiveness of laser-modified cutting tools. Modifications on the flank face were found to have a negligible impact on the process, resulting in a spring back height of less than 4 µm under the specified process conditions. Notably, tools with modifications on the rake face demonstrated substantial reductions in process forces and temperatures for both tested orthogonal setups. Specifically, reductions in the cutting force ranged from 8 to 11 %, and the feed force saw up to 27 %. Additionally, a decrease in temperature on the chip’s free surface of approximately 5 % was observed. Optimal feed rates for a cavity distance $l_{c1'}$ of $\approx$ 34 µm are identified for planing between *f*=0.040 and 0.045 µm and for turning between *f*=0.035 and 0.045 µm. These findings suggest that the contact length of the chip during turning is longer compared to planing. Moreover, the chip formation and development is altered by tool rake face tool modifications showing the tendency to reduce chip segmentations.
3. Simulation setup and results: The study employed SPH simulations, incorporating inversely identified Johnson-Cook parameters, to predict the behavior of a rake face modified tool across various feeds. It is found that the alterations in chip morphology, confirmed by experimental results, are primarily due to the modification’s redirection of chip flow, which changes the effective shear angle, and a decrease in contact length, reducing friction and consequently chip flow resistance. These observations confirm that the simulation accurately reflects experimental outcomes, thereby enriching the understanding of the machining dynamics. SPH simulations accurately captured the cutting force for the reference case, aligning closely with experimental data, with a mean prediction error of about 6.1 % for feeds between 0.015 and 0.050 mm, typically underestimating the forces. Modified tools in the simulations showed even greater precision, with a reduced mean error of approximately 2.6 % for the same feed range. The feed force was $\approx$ 50 % underestimated but shows the impact of the modification at the same feed as in the experiment.
4. Industrial implications: The research suggests that laser-modified tools can be produced at an industrial scale, potentially leading to broader adoption in industries where machining Ti6Al4V is common, such as aerospace and biomedical sectors.

The study concludes that precise tool modifications, using insights from SPH simulations and experimental data, significantly reduce the contact length, process forces, and process temperatures during machining. Overall, the study provides a promising approach to enhancing the machining of difficult-to-cut materials through precise tool modifications, offering significant improvements in machining performance. The successful implementation of these modifications could lead to substantial advancements in machining efficiency, sustainability, and product quality.

## Data Availability

Data will be made available upon request.

## Appendix A: Calculation of rotation axes solutions to align tool surface orthogonal to laser beam

A vector along the cutting edge, $\textbf{k}_n$, and a vector orthogonal to $\textbf{k}_n$ and along the respective tool face, $\textbf{l}_n$, are defined as follows:

A1 $$\begin{aligned} \textbf{k}_n=\begin{pmatrix} k_{nx} \\ k_{ny} \\ k_{nz} \end{pmatrix}, \textbf{l}_n=\begin{pmatrix} l_{nx} \\ l_{ny} \\ l_{nz} \end{pmatrix} \end{aligned}$$

The x and y values of the two vectors are determined by the x and y positions of the touch probe measurements, respectively, which are influenced by the geometry of the tool face. The measurement direction is therefore only in Z-direction resulting in the z values to be the difference between the measurement result of the start and the endpoint of the vectors. As shown in Fig. 1, a rotation around the machine’s B-axis means a rotation around the machine’s Y-axis and can be calculated using the rotation matrix given as follows:

A2 $$\begin{aligned} {{R}_{y}(B)}=\begin{bmatrix} {\cos }(B) &  0 &  {\sin }(B) \\ 0 &  1 &  0 \\ -{\sin }(B) &  0 &  {\cos }(B) \end{bmatrix} \end{aligned}$$

Equivalently, a rotation around the C-axis, in the initial state of B=0$^{\circ }$, is a rotation around the machines Z-axis and can be represented by the following rotation matrix:

A3 $$\begin{aligned} {{R}_{z}(C)}=\begin{bmatrix} {\cos }(C) &  -{\sin }(C) &  0 \\ {\sin }(C) &  {\cos }(C) &  0 \\ 0 &  0 &  1 \end{bmatrix} \end{aligned}$$

A combined rotation around the B-axis and the C-axis can be described by the matrix product of the two rotation matrices:

A4 $$\begin{aligned} \begin{gathered} {R}_{y}(B) \cdot {R}_{z}(C) = \\ \begin{bmatrix} \cos (B) \cdot {\cos }(C) &  -{\cos }(B) \cdot {\sin }(C) &  {\sin }(B) \\ \sin (C) &  \cos (C) &  0 \\ -\sin (B) \cdot \cos (C) &  \sin (B) \cdot \sin (C) &  \cos (B) \end{bmatrix} \end{gathered} \end{aligned}$$

Multiplying this matrix by the vectors $\textbf{k}_n$ and $\textbf{l}_n$ results in the vectors $\textbf{k}_{n+1}$ and $\textbf{l}_{n+1}$, which describe the vectors $\textbf{k}_n$ and $\textbf{l}_n$ after any rotation around B and C, displayed in:

A5 $$\begin{aligned} \textbf{k}_{n+1}= &   (R_y(B) \cdot R_z(C)) \cdot \textbf{k}_n\nonumber \\ \textbf{k}_{n+1}= &   \begin{bmatrix} \cos (B) \cos (C) kx_n - \cos (B) \sin (C) ky_n \\ + \sin (B) kz_n \\ \sin (C) kx_n + \cos (C) ky_n \\ -\sin (B) \cos (C) kx_n + \sin (B) \sin (C) ky_n \\ + \cos (B) kz_n \end{bmatrix}\end{aligned}$$

A6 $$\begin{aligned} \textbf{l}_{n+1}= &   (R_y(B) \cdot R_z(C)) \cdot \textbf{l}_n\nonumber \\ \textbf{l}_{n+1}= &   \begin{bmatrix} \cos (B) \cos (C) lx_n - \cos (B) \sin (C) ly_n \\ + \sin (B) lz_n \\ \sin (C) lx_n + \cos (C) ly_n \\ -\sin (B) \cos (C) lx_n + \sin (B) \sin (C) ly_n \\ + \cos (B) lz_n \end{bmatrix} \end{aligned}$$

For the alignment of the tool face orthogonal to the laser beam, the values of B and C have to be chosen such that the rotated vectors $\textbf{k}_{n+1}$ and $\textbf{l}_{n+1}$ must lie in the x-y plane, which is achieved when their Z-components are zero, resulting in the following system of equations:

A7 $$\begin{aligned} {\left\{ \begin{array}{ll} \begin{aligned} & -\sin (B) \cos (C) kx_n + \sin (B) \sin (C) ky_n \\ & + \cos (B) kz_n = 0 \end{aligned} \\ \begin{aligned} & -\sin (B) \cos (C) lx_n + \sin (B) \sin (C) ly_n \\ & + \cos (B) lz_n = 0 \end{aligned} \end{array}\right. } \end{aligned}$$

The solution of Eq. A7 for B and C is given in Section 2.3 in Eqs. 1 and 2.

## Appendix B: Tabular force and temperature values

**Table 7:** $F_c$, $F_f$ and *T* for reference tool of planing experiment

Exp	Mod	Feed	$F_c$	$F_f$	*T*
		[mm]	[N/mm]	[N/mm]	[°C]
1076	Ref	0.010	29.5	27.0	296.5
1077	Ref	0.015	37.7	29.6	325.5
1078	Ref	0.020	46.5	31.0	382.8
1079	Ref	0.025	54.4	31.8	390.5
1080	Ref	0.030	61.6	32.0	401.2
1081	Ref	0.035	69.4	32.4	410.4
1082	Ref	0.040	76.5	32.2	416.5
1083	Ref	0.045	83.7	31.8	421.9
1084	Ref	0.050	90.9	31.6	426.2

**Table 8:** $F_c$, $F_f$ and *T* for flank face modified tool of planing experiment

Exp	Mod	Feed	$F_c$	$F_f$	*T*
		[mm]	[N/mm]	[N/mm]	[°C]
1066	FF	0.010	20.0	20.0	−
1067	FF	0.015	38.1	30.3	335.6
1068	FF	0.020	46.7	32.3	379.0
1069	FF	0.025	53.9	32.2	392.5
1070	FF	0.030	61.2	33.0	−
1071	FF	0.035	68.9	33.8	413.5
1072	FF	0.040	75.9	33.5	420.1
1073	FF	0.045	83.2	33.7	424.9
1075	FF	0.050	90.5	33.5	430.2

**Table 9:** $F_c$, $F_f$ and *T* for rake face modified tool of planing experiment

Exp	Mod	Feed	$F_c$	$F_f$	*T*
		[mm]	[N/mm]	[N/mm]	[°C]
1057	RF	0.010	26.8	24.6	320.8
1058	RF	0.015	37.4	28.5	345.8
1059	RF	0.020	44.6	28.5	376.2
1060	RF	0.025	50.9	27.4	385.5
1061	RF	0.030	56.7	26.5	389.7
1062	RF	0.035	63.0	25.3	396.8
1063	RF	0.040	68.8	24.3	400.8
1064	RF	0.045	74.9	23.4	401.4
1065	RF	0.050	81.3	23.2	402.9

**Table 10:** $F_c$ and $F_f$ for reference tool of turning experiment

Exp	Mod	Feed	$F_c$	$F_f$
		[mm]	[N/mm]	[N/mm]
1100	Ref	0.010	33.0	27.4
1101	Ref	0.015	42.2	28.1
1102	Ref	0.020	50.3	28.5
1103	Ref	0.025	58.2	28.8
1104	Ref	0.030	66.5	29.7
1105	Ref	0.035	73.5	29.6
1106	Ref	0.040	81.2	30.1
1107	Ref	0.045	88.2	30.0
1108	Ref	0.050	95.0	29.9

**Table 11:** $F_c$ and $F_f$ for rake face modified tool of turning experiment

Exp	Mod	Feed	$F_c$	$F_f$
		[mm]	[N/mm]	[N/mm]
1091	RF	0.010	−	−
1092	RF	0.015	39.9	23.5
1093	RF	0.020	46.7	22.5
1094	RF	0.025	53.4	21.4
1095	RF	0.030	61.2	22.2
1096	RF	0.035	67.9	21.7
1097	RF	0.040	75.1	21.3
1098	RF	0.045	83.0	22.5
1099	RF	0.050	−	−
